# Behavior-Based Screening in Organ and Tissue Donor Risk Assessments in Two-Spirit, Gay, Lesbian, Bisexual, Transgender, Queer, Intersex and Other Sexual and Gender Minority (2S/LGBTQI+) People: A Canada-wide Community Survey

**DOI:** 10.1177/20543581261474939

**Published:** 2026-09-30

**Authors:** Murdoch Leeies, Kimia Rohani, David Collister, Karen Doucette, Emily Christie, Carmen Hrymak, Sonny Dhanani, Warren Fingrut, Tzu-Hao Lee, Ken Sutha, Cameron T. Whitley, Ben Klassen, Nathan Lachowsky

**Affiliations:** 1 Department of Emergency Medicine, University of Manitoba, Winnipeg, Canada; 2 Transplant Manitoba, Winnipeg, Canada; 3 584047 Community Based Research Centre, Vancouver, Canada; 4 Department of Medicine, University of Alberta, Edmonton, Canada; 5 6363 Department of Pediatrics, University of Ottawa, Ottawa, Canada; 6 Children’s Hospital of Eastern Ontario, Ottawa, Canada; 7 Department of Stem Cell Transplantation and Cellular Therapy, 731302 The University of Texas MD Anderson Cancer Center, Houston, TX, USA; 8 3989 Department of Gastroenterology, Baylor College of Medicine, Houston, USA; 9 Department of Medicine, Stanford University, Stanford, USA; 10 1632 Faculty of Sociology, Western Washington University, Bellingham, USA; 11 Department of Public Health and Social Policy, University of Victoria, Victoria, Canada; 12 6727 Faculty of Human and Health Sciences, University of Northern British Columbia, Prince George, Canada

**Keywords:** deceased organ donor registration, gender, Living kidney donation, organ donation, transplantation

## Abstract

**Purpose:**

Two-Spirit, Lesbian, Gay, Bisexual, Transgender, Queer, Intersex and other sexually and gender diverse (2S/LGBTQI+) populations experience discriminatory donor risk assessments in the Canadian organ and tissue donation (OTD) system. Based on identity and/or sexual behavior many 2S/LGBTQI+ people are deemed increased infectious risk donors under current regulations. We sought to: 1) understand the predictive ability of newly updated sexual behavior-based screening in both deceased and living donation, 2) describe 2S/LGBTQI+ community perspectives on newly proposed Health Canada donor risk assessment criteria, and 3) identify 2S/LGBTQI+ community priorities in OTD system improvement through a national survey.

**Methods:**

We conducted a cross-sectional, web-based Canada-wide survey of 2S/LGBTQI+ people in Canada (April 16 – June 11, 2025). Participant characteristics were summarized using descriptive statistics. The likelihood of different substitute decision makers to answer sexual behavior-based screening questions was assessed with bivariate analyses, data are presented as counts and row percentages to illustrate the distribution of outcomes within each predictor group. Continuous variables are presented as means with standard deviations (SD) and ranges.

**Results:**

We analyzed responses from 2072 respondents. Participants reported heterogeneity in the likelihood that their medical decisionmakers would accurately answer questions about their sexual behaviors, sexual partners, substance use, and HIV Pre- or Post-Exposure Prophylaxis (PrEP/PEP) use, which particularly depended on who their medical decision maker was (e.g. a partner or friend versus other family). Those at highest risk for HIV were less likely to report that their medical decisionmakers would accurately answer questions about their sexual partners. Approximately 20% of survey participants reported they were uncertain or would not self-report these details if they were directly asked by healthcare providers. More than half of participants felt the newly proposed Health Canada donor risk assessment criteria were discriminatory and required further change.

**Conclusions:**

Sexual behavioral screening for donor risk assessments in 2S/LGBTQI+ people living in Canada is likely to be poorly predictive of the risk of HIV and viral hepatitis transmission through organ and tissue transplantation. Health Canada should support a re-evaluation of the current increased infectious risk donor paradigm guided by evidence and patient/community values/preferences to maximize benefits and minimize harms to patients and to 2S/LGBTQI+ community members.

## Background

Numerous inequities for Two-Spirit, Lesbian, Gay, Bisexual, Transgender, Queer, Intersex and other sexually and gender diverse (2S/LGBTQI+) populations in the Canadian organ and tissue donation and transplantation (OTDT) system have been identified from academic, community, patient and caregiver perspectives.^1-9^ Researchers and 2S/LGBTQI+ community-based advocates have called for equitable policy revision from Health Canada and identified specific targets for action.^4,10^ In Canada, demand for transplantable organs and tissues far exceeds available supply, resulting in ongoing morbidity and preventable deaths among individuals waiting for transplantation.^11-13^ However, many members of the 2S/LGBTQI+ community have been considered at increased risk of transmitting infections through donation based on their identity rather than actual behavioral risk.^4,14-18^ This has limited how they can donate organs and prevents them from also donating tissues. The risk of HIV acquisition is heterogeneous within 2S/LGBTQI+ populations. The HIV Incidence Risk Index for Men who have Sex with Men (HIRI-MSM) is a validated clinical prediction tool developed to estimate a person’s risk of HIV acquisition and used to identify those who may benefit from HIV pre-exposure prophylaxis (PrEP). We utilized HIRI-MSM as a validated measure of HIV acquisition risk to understand whether perceptions regarding behavior-based donor screening varied according to underlying HIV risk.^the re^

A recent survey of 2S/LGBTQI+ people living in Canada found the majority (79%) wanted to donate their organs and tissues but were unaware of discriminatory policies that impacted them.^9^ Every potential donor must be tested for Human Immunodeficiency Virus (HIV) and viral hepatitis before donating, but in the very rare event the test is inaccurate, donors are still classified as baseline versus increased risk based on a list of risk factors.^19,20^ In September 2025, Health Canada published new donor risk assessment standards that focus on gender-neutral, behavior-based screening, rather than identity-based screening (Figure 1).^21,22^

**Figure 1.**
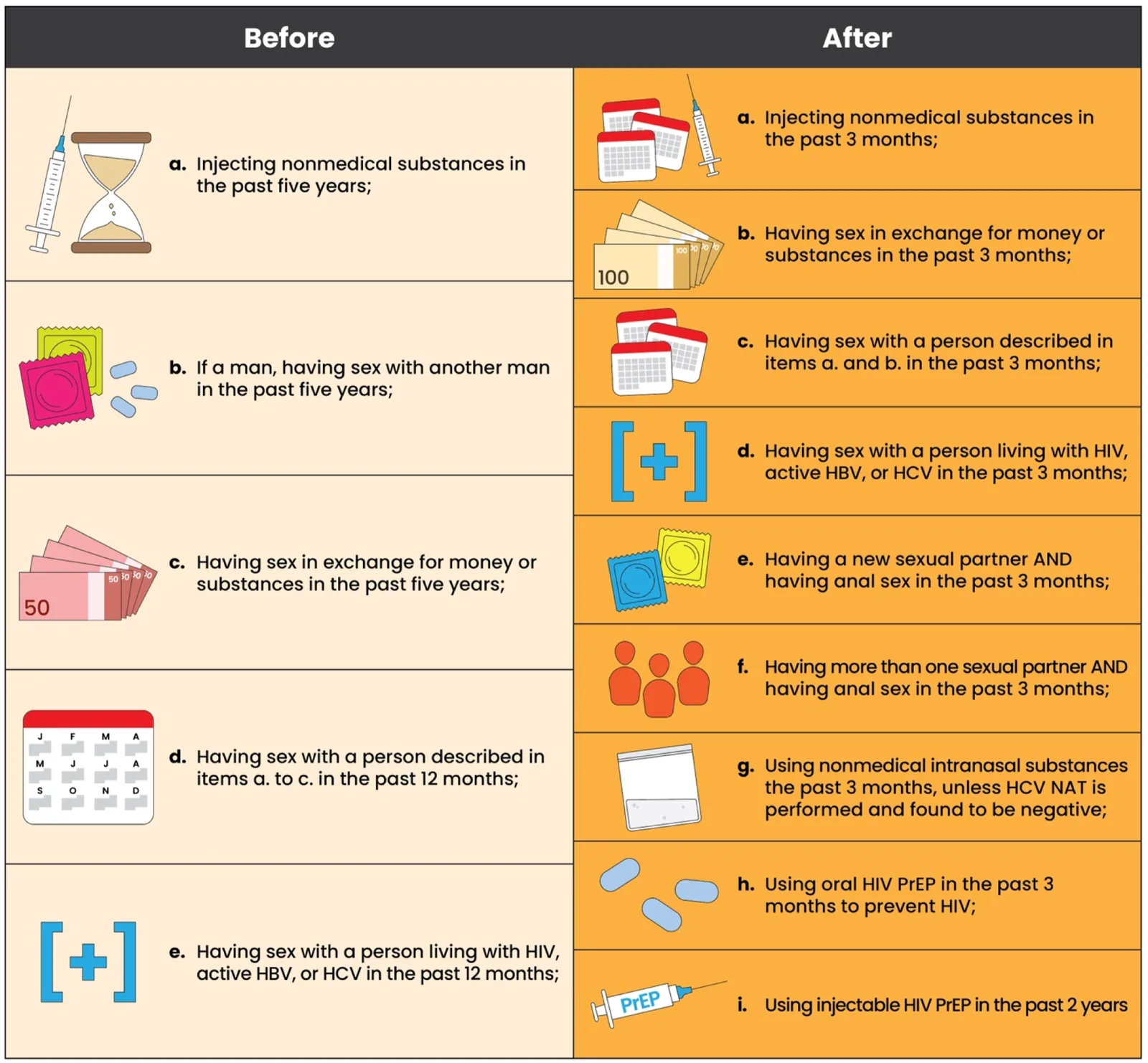
Health Canada donor risk assessment changes

In deceased organ donation, behavior-based screening of the potential donor relies on questions about their sex life, non-medical injection drug use, and other risk factors for HIV and hepatitis virus acquisition. These questions are typically directed to the potential donor’s next-of-kin. The accuracy of behavior-based screening via self-report (only possible in living donation) versus secondary history-taking from next-of-kin (the only option available in deceased donation) in 2S/LGBTQI+ populations has not been evaluated. We sought to: 1) understand the predictive ability of sexual behavior-based screening in both deceased and living donation to inform updated criteria, 2) describe 2S/LGBTQI+ community perspectives on newly proposed donor risk assessment criteria, and 3) identify 2S/LGBTQI+ community priorities in OTDT system improvement in this national survey.

## Methods

### Study Design

We performed a web-based, cross-sectional survey, administered as a module within the Community Based Research Centre’s (CBRC’s) ongoing *Sex Now* Canada-wide survey of 2S/LGBTQI+ people in Canada.^9,23^

### Questionnaire

Questionnaire design was informed by the Dillman methodology.^24^ Questions focused on single constructs with an emphasis on accessible and concise response items. We generated items iteratively informed by literature review and discussion with collaborators and a pre-existing group of 2S/LGBTQI+ patient/caregiver partners with lived experience of the organ and tissue donation system. This team pilot tested the questionnaire for readability, comprehension, content-validity and comprehensiveness prior to distribution. The questionnaire was professionally translated into French and Spanish by 2S/LGBTQI+-competent professional translators, with additional review through CBRC team members. All translations were pilot tested by multiple people for accuracy and appropriateness of terminology (Appendix 1).

At the time of survey completion, new behavioral, gender neutral donor risk assessment questions were proposed by Health Canada for public feedback. After survey completion Health Canada accepted and formalized these criteria (September 2025) which were being incorporated into clinical practice across Canada at the time of writing this manuscript.^22^

### Survey Administration

We distributed our module in the *Sex Now* 2025 survey cycle, which was open for recruitment from April 16 – June 11, 2025. Participation was anonymous. Details of *Sex Now* survey methodology have been previously published.^9,23^ This ongoing, cross-sectional survey has been conducted by the CBRC since 2002 and aims to promote the health and wellness of 2S/LGBTQI+ people living in Canada. Participants for this iteration were recruited via social media where the CBRC has an established network, the CBRC’s newsletter, membership and through community-based partner organizations across Canada and using social and sexual networking apps and websites (e.g., Squirt, Scruff, HER). Eligibility requirements included age 15 or older, currently living in Canada, and able to complete an online survey in French, English or Spanish. Participants had to identify as 2S/LGBTQI+ or another non-heterosexual or non-cisgender identity or be a man who had sex with another man in the past five years. Informed consent was documented in Qualtrics survey platform from each participant prior to questionnaire self-completion. Potential duplicate responses were identified based on device- and browser-level metadata and removed following manual review. To further minimize bot and fraudulent respondents, we offered participants a prize draw incentive (one of three $500 travel vouchers) instead of a direct cash honorarium. Complete responses were not mandatory to progress through the survey.

### Statistical Analysis

Participant characteristics were summarized using descriptive statistics. In Table 1, categorical variables are presented as counts and column percentages to describe the total study population. In subsequent bivariate analyses (Table 2), data are presented as counts and row percentages to illustrate the distribution of outcomes within each predictor group. Continuous variables are presented as means with standard deviations (SD) and ranges, as appropriate.

**Table 1.** Baseline Characteristics of Sex now 2025 Online Survey Participants (N=2,072)

Characteristic	n (%)
Age groups (n = 2,072)	
15-19 years old	49 (2.4%)
20-29 years old	379 (18%)
30-39 years old	534 (26%)
40-49 years old	404 (19%)
50-59 years old	322 (16%)
60+ years old	384 (19%)
Language of Survey Completion (n = 2,072)	
English	1,858 (90%)
French	200 (9.7%)
Spanish	14 (0.7%)
Province/Territory of Residence (n = 2,072)	
Alberta	226 (11%)
British Columbia	478 (23%)
Manitoba	76 (3.7%)
New Brunswick	51 (2.5%)
Newfoundland & Labrador	27 (1.3%)
Northwest Territories	1 (<0.1%)
Nova Scotia	108 (5.2%)
Ontario	779 (38%)
Prince Edward Island	10 (0.5%)
Québec	273 (13%)
Saskatchewan	41 (2.0%)
Yukon Territory	2 (<0.1%)
Race/Ethnicity, Single Group (n = 2,072)	
Black	58 (2.8%)
East Asian	43 (2.1%)
Indigenous	58 (2.8%)
Latina, Latino, Latinx, Latine	71 (3.4%)
Southwest Asian and North Africa	20 (1.0%)
South Asian	64 (3.1%)
Southeast Asian	23 (1.1%)
White	1,498 (72%)
Multiracial	165 (8.0%)
None	72 (3.5%)
Are you Two-Spirit? (n = 137^†^)	
I am Two-Spirit	64 (47%)
I am not Two-Spirit	60 (44%)
Prefer not to answer	13 (9.5%)
Sexual Orientation* (n = 2,068)	
Asexual	80 (3.9%)
Bisexual	616 (30%)
Demisexual	107 (5.2%)
Gay	1,107 (54%)
Heteroflexible	53 (2.6%)
Homoflexible	73 (3.5%)
Lesbian	154 (7.4%)
Pansexual	241 (12%)
Queer	604 (29%)
Questioning	40 (1.9%)
Straight	53 (2.6%)
Other	55 (2.7%)
Missing	4
Openness: Sexual orientation (n = 2,057)	
1-Not at all open	190 (9.2%)
2	183 (8.9%)
3	266 (13%)
4	375 (18%)
5-Open (out) to all or most people	1,043 (51%)
Missing	15
Do you identify as trans? (n = 2,072)	
Yes	334 (16%)
Unsure or questioning	74 (3.6%)
No	1,664 (80%)
What was your sex assigned at birth? (n = 2,072)	
Female	565 (27%)
Male	1,493 (72%)
Prefer not to answer	14 (0.7%)
Are you an intersex person (n = 2,068)	
Yes	44 (2.1%)
No	1,906 (92%)
Unsure	118 (5.7%)
Missing	4
Man (cis or trans) who had sex with a man (cis or trans) in the past 12 months (n = 2,069)	
Yes	1,151 (56%)
No	918 (44%)
Missing	3
Gender Identity* (n = 2,069)	
Agender	40 (1.9%)
Genderfluid	91 (4.4%)
Genderqueer	144 (7.0%)
Man	1,396 (67%)
Non-Binary	273 (13%)
Questioning	48 (2.3%)
Trans man	99 (4.8%)
Trans woman	45 (2.2%)
Transfeminine	41 (2.0%)
Transmasculine	95 (4.6%)
Woman	299 (14%)
Other	54 (2.6%)
Missing	3
Openness: Gender Identity (n = 2,050)	
1-Not at all open	142 (6.9%)
2	119 (5.8%)
3	173 (8.4%)
4	204 (10.0%)
5-Open (out) to all or most people	1,412 (69%)
Missing	22
Do you identify as a person with a disability? (n = 2,068)	
Yes	521 (25%)
No	1,440 (70%)
Unsure	107 (5.2%)
Missing	4
Overall, how would you describe your money situation in the past 6 months? (n = 2,068)	
Cannot make ends meet	224 (11%)
Have to cut back	443 (21%)
Enough, but no extra	697 (34%)
Comfortable, with extra	704 (34%)
Missing	4
What best describes the environment you live in? (n = 2,065)	
Rural area (Less than 1,000 people)	105 (5.1%)
Small population centre (1,000-29,999 people)	208 (10%)
Medium population centre (30,000-99,999 people)	275 (13%)
Large urban population centre (100,000-499,999 people)	421 (20%)
Very large urban population centre (500,000+ people)	1,056 (51%)
Missing	7
What is the highest level of education that you have completed? (n = 2,065)	
Did not finish high school	71 (3.4%)
High school or equivalent	334 (16%)
Post-secondary school (e.g. certificate, diploma, CEGEP)	517 (25%)
Bachelor’s degree	630 (31%)
Above a bachelor’s degree (e.g. masters, doctorate)	513 (25%)
Missing	7
HIRI-MSM Score (n = 1,395^#^)	
7 ± 10 (0-45) Mean ± SD (Min-Max)	7 ± 10 (0-45)
Have you EVER taken HIV PrEP (n = 1,382^#^)	
Yes, I am currently taking HIV PrEP	347 (25%)
Yes, but I stopped in the past 6 months	33 (2.4%)
Yes, but I stopped more than 6 months ago	70 (5.1%)
No	932 (67%)
Missing	24
Would you be willing to donate* (n = 1,753)	
Organs after my death	1,418 (81%)
Tissues after my death	1,311 (75%)
Organs as a living organ donor	713 (41%)
Stem cells as a living donor	948 (54%)
None of the above	266 (15%)
Missing	319

HIRI-MSM: HIV Incidence Risk Index-Men who have Sex with Men; PrEP: HIV Pre-Exposure Prophylaxis.

*Multiple responses were permitted; therefore, percentages do not sum to 100%.

†This question was displayed only to respondents identified as Indigenous.

^#^Displayed only to respondents reporting HIV-negative status or non-response to the HIV diagnosis question.

**Table 2.** Behavior-Based Screening via Substitute Decision-Maker Among Sex now 2025 Online Survey Participants

​	How likely is it that your medical decisionmaker would accurately answer questions about your recent sex life?			Row total (N^#^)	p-value^†^
	Very/Somewhat unlikely	Neither likely nor unlikely	Very/Somewhat likely		
**Current Medical Decision Maker (Merged categories)**					<0.001
My partner	80 (14%)	20 (3.4%)	491 (83%)	591	​
Family member (Immediate/Extended)	300 (50%)	53 (8.8%)	247 (41%)	600	​
Someone else I have chosen as my medical decision maker (e.g. friend)	34 (23%)	10 (6.8%)	104 (70%)	148	​
I do not know	173 (48%)	74 (21%)	113 (31%)	360	​
**HIRI-MSM score** *					0.058
Less than 10	236 (32%)	55 (7.5%)	442 (60%)	733	​
Greate than or equal to 10	239 (37%)	61 (9.3%)	353 (54%)	653	​
​	How likely is it that your medical decisionmaker would accurately answer questions about your recent sexual partner(s)?			​	​
​	**Very/Somewhat unlikely**	**Neither likely nor unlikely**	**Very/Somewhat likely**	**Row Total (N**^#^)	**p-value** ^†^
**Current Medical Decision Maker (Merged categories)**					<0.001
My partner	112 (19%)	40 (6.8%)	437 (74%)	589	​
Family member (Immediate/Extended)	358 (60%)	55 (9.2%)	187 (31%)	600	​
Someone else I have chosen as my medical decision maker (e.g. friend)	52 (35%)	24 (16%)	73 (49%)	149	​
I do not know	190 (53%)	68 (19%)	100 (28%)	358	​
**HIRI-MSM score** *					<0.001
Less than 10	280 (38%)	67 (9.2%)	384 (53%)	731	​
Greate than or equal to 10	298 (46%)	81 (12%)	273 (42%)	652	​
​	How likely is it that your medical decision maker would accurately answer questions about your recent substance use?			​	​
​	**Very/Somewhat unlikely**	**Neither likely nor unlikely**	**Very/Somewhat likely**	**Row Total (N**^#^)	**p-value** ^†^
**Current Medical Decision Maker (Merged categories)**					<0.001
My partner	56 (9.5%)	17 (2.9%)	517 (88%)	590	​
Family member (Immediate/Extended)	199 (33%)	36 (6.0%)	365 (61%)	600	​
Someone else I have chosen as my medical decision maker (e.g. friend)	39 (26%)	11 (7.4%)	98 (66%)	148	​
I do not know	141 (39%)	63 (18%)	154 (43%)	358	​
**HIRI-MSM score**					0.9
Less than 10	177 (24%)	51 (7.0%)	503 (69%)	731	​
Greate than or equal to 10	164 (25%)	48 (7.4%)	440 (67%)	652	​
​	How likely is it that your medical decision maker would accurately answer questions about your recent use of HIV PrEP or PEP?			​	​
​	**Very/Somewhat unlikely**	**Neither likely nor unlikely**	**Very/Somewhat likely**	**Row Total (N**^#^)	**p-value** ^†^
**Current Medical Decision Maker (Merged categories)**					<0.001
My partner	68 (12%)	23 (3.9%)	497 (85%)	588	​
Family member (Immediate/Extended)	265 (44%)	70 (12%)	265 (44%)	600	​
Someone else I have chosen as my medical decision maker (e.g. friend)	34 (23%)	19 (13%)	96 (64%)	149	​
I do not know	150 (42%)	78 (22%)	129 (36%)	357	​
**HIRI-MSM score** *					0.095
Less than 10	210 (29%)	71 (9.7%)	450 (62%)	731	​
Greate than or equal to 10	210 (32%)	77 (12%)	364 (56%)	651	​

HIRI-MSM: HIV Incidence Risk Index-Men who have Sex with Men; PrEP: Pre-Exposure Prophylaxis, PEP: Post-Exposure Prophylaxis.

*Participants with a pre-existing HIV-positive diagnosis were excluded from this specific sub-analysis.

#N represents the analytic sample (Denominator) for each row. Percentage values are row percentages, indicating the distribution of likelihood within each decision-maker or HIRI category.

†Statistical significance was determined using Pearson’s Chi-square tests.

To ensure statistical power and address sparse cell counts (specifically for the ‘My child’ and ‘Extended family’ categories), the ‘Medical Decision Maker’ variable was collapsed into four logically grouped cohorts: Partner, Family member (Immediate/Extended), Chosen Friend/Other, and I do not know. Friends/Other were maintained as a standalone category due to a distinct distribution of perceived accuracy compared with relatives. Inferential analyses were performed using Pearson’s Chi-square tests to assess the association between decision-maker types, HIRI-MSM score categories, and the four accuracy-related outcome variables.

The HIRI-MSM score analysis was restricted to a sub-sample of participants who reported being HIV-negative or of unknown status. To verify the robustness of the associations, sensitivity analyses were performed by excluding the ‘I do not know’ category. These analyses confirmed that the exclusion did not substantively alter the statistical significance or direction of the findings.

Missing data were handled using available-case analysis; denominators reflect non-missing responses for each specific test. All analyses were conducted using R version 4.5.1. Reporting is consistent with the CROSS checklist.^25^

### Ethics

Harmonized research ethics approval was received from the University of Victoria, University of British Columbia, and Simon Fraser University (H23-02083). Funding organizations had no role in survey design, implementation or analysis.

## Results

We received and analyzed 2072 survey responses from 2S/LGBTQI+ community members across Canada. Of these, 3% (64/2072) self-identified as Indigenous Two-Spirit, 7% (154/2072) Lesbian, 54% (1107/2072) Gay, 30% (616/2072) Bisexual, 16% (334/2072) Transgender, 29% (604/2072) Queer, and 2% (44/2072) Intersex. In terms of race/ethnicity, 16% (324/2072) identified as racialized and 7% (140/2072) as Indigenous. Most participants reported having sex with a man in the past 12 months (70%, 1460/2072). Of HIV-negative participants, 33% (450/1382) reported currently or previously taking HIV pre-exposure prophylaxis (PrEP). The mean HIV Incidence Risk Index (HIRI-MSM) score^26,27^ was 7 (SD +/- 10), noting that a score of >10 suggests a clinical indication for PrEP.^26,27^ Participants completed the survey in English (90%, 1858/2072), French (10% (200/2072), and Spanish (1%, 14/2072). (Table 1).

### Behavior-Based Screening via Substitute Decisionmaker

Participants were asked to indicate their decisionmaker in the event they were unable to make their own medical decisions, which was either their partner (35%, 595/1722), parent (22%, 385/1722), friend (9%, 153/1722), sibling (8%, 130/1722), or child (2%, 41/1722) (Figure 2). Most who named a partner decisionmaker said their partner would be very or somewhat likely to accurately answer questions about their recent sex life (83%, 491/591) and sexual partners (74%, 437/589); however, other categories of decisionmakers were reported as being less likely to know accurately about sexual behaviors or sexual partners (all statistically significant at p<0.001, Table 2). Those with a higher risk of HIV acquisition based on HIRI-MSM score (HIRI score >/= 10) were less likely to report that their medical decisionmaker would be able to accurately answer questions (compared with participants with lower HIV risk) about their recent sex life (54%, 353/653 versus 60%, 442/733, p=0.058), sexual partners (42%, 273/652 versus 53%, 384/731, p<0.001), substance use (67%, 440/652 versus 69%, 503/731, p=0.900) or PrEP use (56%, 364/651 versus 62%, 450/731 p=0.095) (Table 2). The perceived likelihood that a participant’s medical decision maker would accurately answer relevant behavior-based screening questions are visually summarized in Figure 3.

**Figure 2.**
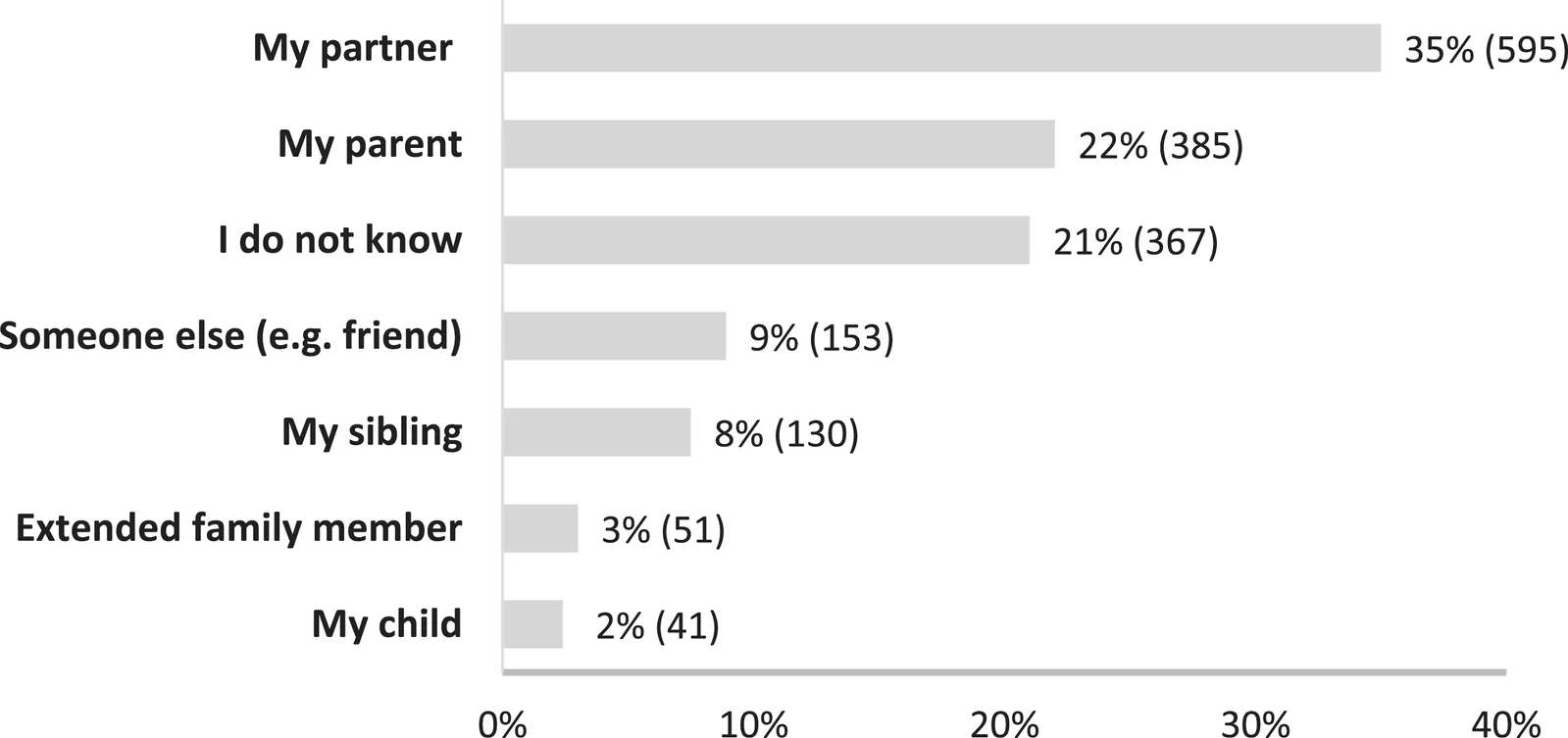
Current medical decision-maker among Sex Now 2025 online survey participants. Percentages are calculated among participants with non-missing responses (n = 1,722). Missing responses (n = 350) are not shown

**Figure 3.**
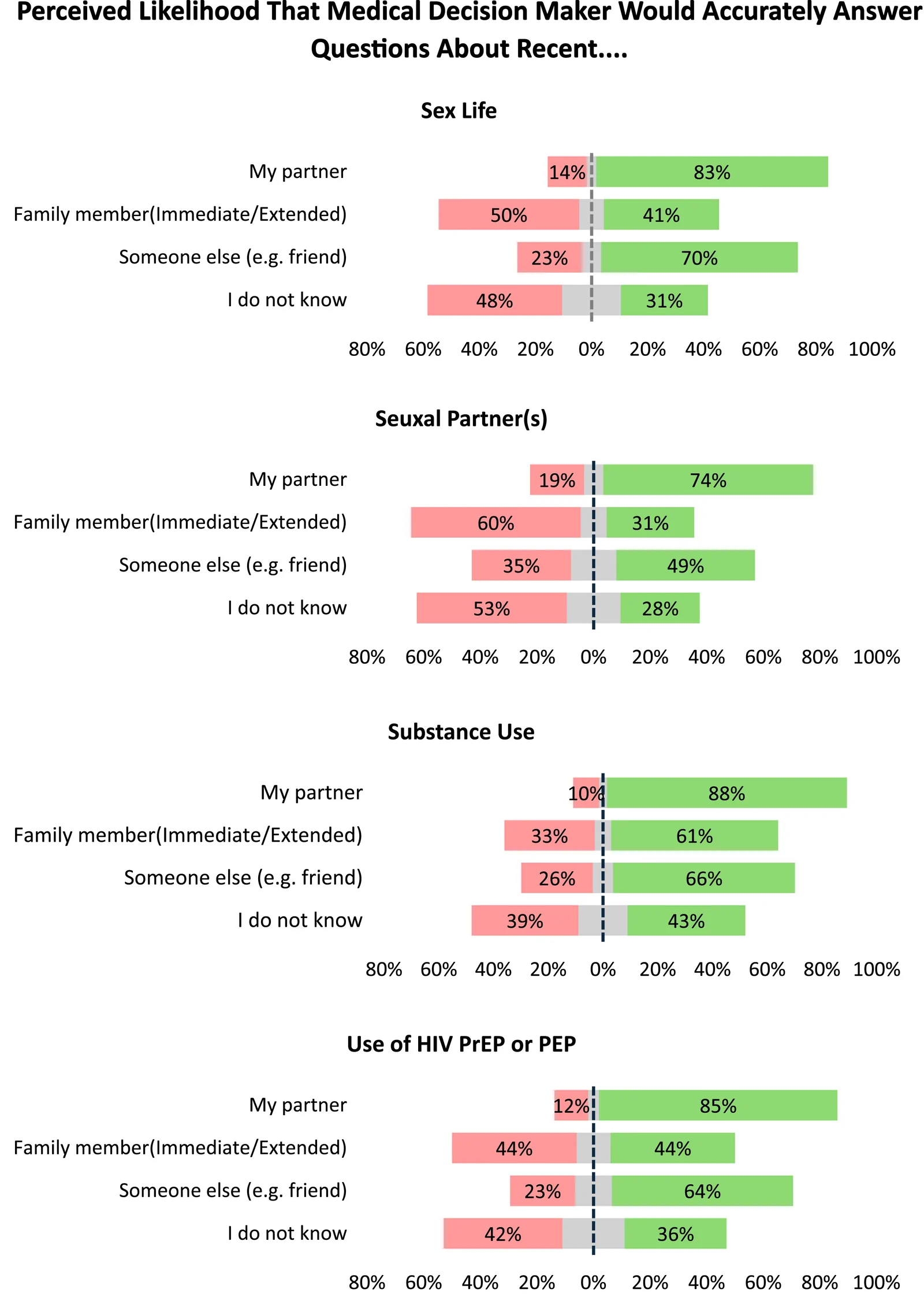
Behavior-based screening via medical decision Maker Perceived likelihood that medical decision maker would accurately answer questions about recent

### Behavior-Based Screening via Self-Report

Most participants reported that they would answer accurately if their transplant team asked about their recent sex life (77%, 1274/1656), sexual partners (75%, 1245/1654), substance use (81%, 1338/1646), and HIV PrEP/PEP use (79%, 1300/1640). However, when asked about trust in the health system only 62% (1024/1657) said that they trusted the health system enough to provide accurate responses to their transplant teams for these questions (Figure 4).

**Figure 4.**
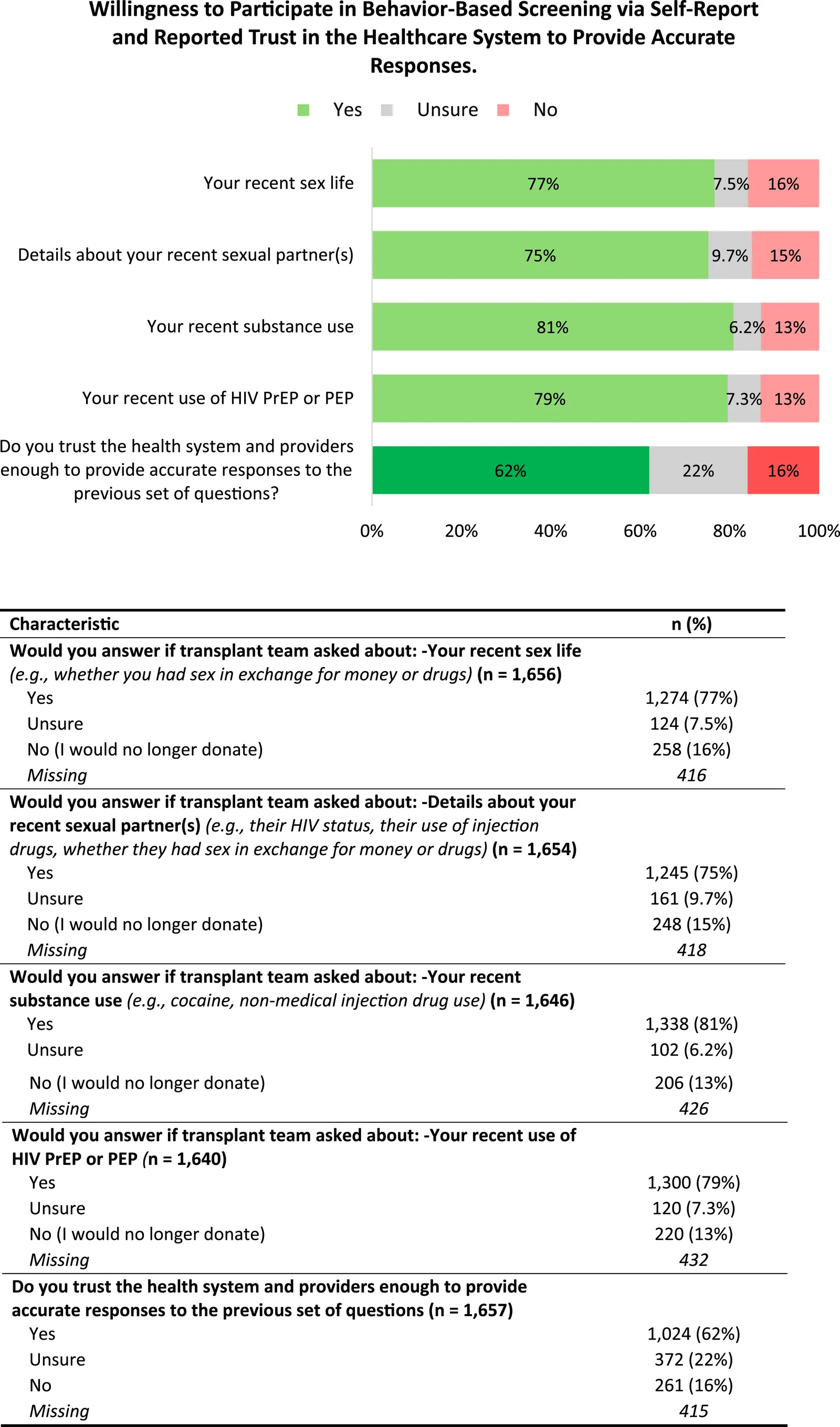
Behavior-based screening via self-report

### Likelihood of Answering New Proposed Criteria

In response to newly proposed gender-neutral, behavior-based potential questions to replace previous identity-based donor risk assessment criteria, 81% (1505/1863) of participants said they would answer accurately if asked whether they had sex with a new partner in the last 3 months, 77% (1440/1860) if asked whether they had sex with more than 1 partner in the last 3 months, and 72% (1340/1859) if asked whether they had anal sex in the last 3 months. If asked about HIV PrEP/PEP use, 26% (480/1863) said they would not answer and would no longer donate (Figure 5).

**Figure 5.**
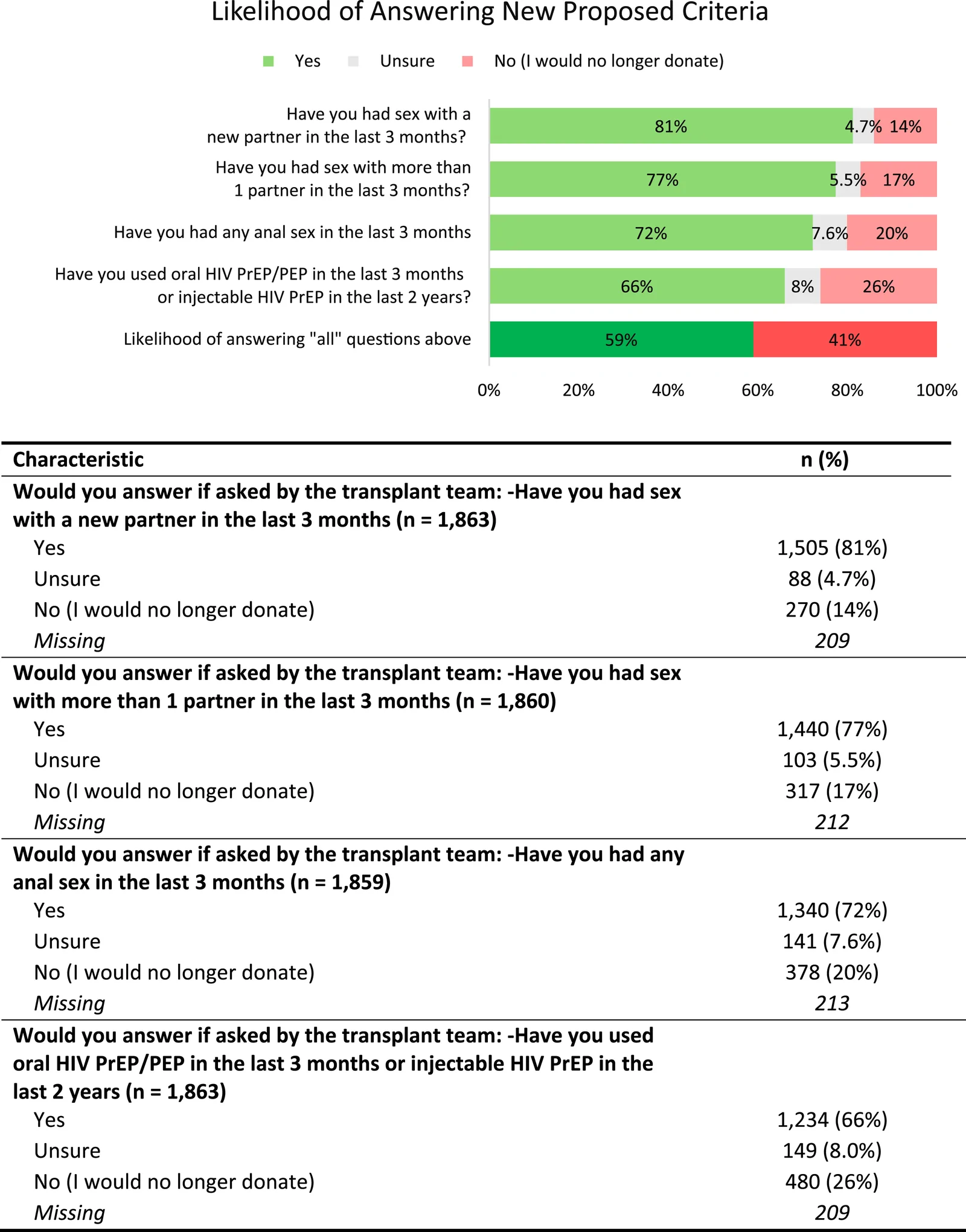
Likelihood of answering new proposed criteria

### Agreement With Proposed Policy Revision

Just over half of participants (51%, 905/1753) agreed that the newly proposed Health Canada organ and tissue donation policy changes were discriminatory, with nearly a third (29%, 508/1753) neutral. When asked if the policy changes were justified, 39% (693/1762) agreed, 30% (531/1762) were neutral, and 30% (538/1762) disagreed. Most participants (64%, 1122/1756) agreed the proposed policy updates require further change, while only 7% (114/1756) disagreed (Table 3).

**Table 3.** Level of Agreement With Proposed Policy Revision by Sex now 2025 Online Survey Participants

Characteristic	n (%)
The proposed Health Canada organ and tissue donation policy is discriminatory (n = 1,753)	
Strongly agree	357 (20%)
Somewhat agree	548 (31%)
Neither agree nor disagree	508 (29%)
Somewhat disagree	176 (10%)
Strongly disagree	164 (9.4%)
Missing	319
The proposed Health Canada organ and tissue donation policy is justified (n = 1,762)	
Strongly agree	254 (14%)
Somewhat agree	439 (25%)
Neither agree nor disagree	531 (30%)
Somewhat disagree	343 (19%)
Strongly disagree	195 (11%)
Missing	310
The proposed Health Canada organ and tissue donation policy requires further change (n = 1,756)	
Strongly agree	548 (31%)
Somewhat agree	574 (33%)
Neither agree nor disagree	520 (30%)
Somewhat disagree	65 (3.7%)
Strongly disagree	49 (2.8%)
Missing	316
Health Canada should not consider sexual contact with people living with HIV who are undetectable as increasing risk for the HIV-negative partner (n = 1,747)	
Strongly agree	520 (30%)
Somewhat agree	431 (25%)
Neither agree nor disagree	497 (28%)
Somewhat disagree	191 (11%)
Strongly disagree	108 (6.2%)
Missing	325
Health Canada should not consider recent use of HIV PEP or PrEP as increased risk in organ donation (n = 1746)	
Strongly agree	583 (33%)
Somewhat agree	465 (27%)
Neither agree nor disagree	471 (27%)
Somewhat disagree	148 (8.5%)
Strongly disagree	79 (4.5%)
Missing	326
The proposed Health Canada organ and tissue donation policy is based on the best available research evidence (n = 1,756)	
Strongly agree	270 (15%)
Somewhat agree	424 (24%)
Neither agree nor disagree	611 (35%)
Somewhat disagree	275 (16%)
Strongly disagree	176 (10%)
Missing	316

HIV: human immune deficiency virus, PEP: post-exposure prophylaxis, PrEP: pre-exposure prophylaxis

Regarding specific policy change options, over half (55%, 951/1747) agreed that Health Canada should not consider sexual contact with people living with HIV who are undetectable as increasing risk for the HIV-negative partner, while 28% (497/1747) were unsure and 17% (299/1747) disagreed. Most (60%, participants also agreed that Health Canada should not consider recent HIV PrEP/PEP use as increased risk (1048/1746), while 27% (471/1746) were unsure and 13% (227/1746) disagreed (Table 3).

#### Priorities for Policy Action

Most participants endorsed Health Canada implementing more precise screening for sexual practices to allow more 2S/LGBTQI+ people to donate (73%, 1110/1531), improved sexual and gender diversity training for donation staff (68%, 1041/1531), 2S/LGBTQI+ specific outreach efforts (55%, 847/1531), and promotion of donation options by 2S/LGBTQI+ community organizations (56%, 853/1531). Most said Canadian OTDT regulators and operators should make reparations to the 2S/LGBTQI+ community for existing policies (54%, 839/1551), or weren’t sure (24%, 366/1551). Most (62%, 990/1602) agreed with increased transparency about restrictions on organ and tissue donation that impact 2S/LGBTQI+ people with active disclosure to potential donors (Table 4).

**Table 4.** Priorities for Policy Action Among Sex now 2025 Online Survey Participants

Characteristic	n (%)
Additional organ and tissue donation changes* (n = 1,531)	
More precise screening for sexual practices to allow more 2S/LGBTQI+ people to donate	1,110 (73%)
Improved sexual and gender diversity training for donation staff	1,041 (68%)
2S/LGBTQI+-specific outreach efforts	847 (55%)
Promotion of donation options by 2S/LGBTQI+ community organizations	853 (56%)
Other	95 (6.2%)
None of the above	178 (12%)
Missing	541
Do you believe that Health Canada, Canadian Blood Services and Transplant Québec should apologize and make reparations to the 2S/LGBTQI+ community for existing organ and tissue donation policies? (n = 1,551)	
Yes	839 (54%)
I am not sure	366 (24%)
No	346 (22%)
Missing	521
Level of agreement with the following statement: “Health Canada, Canadian Blood Services and Transplant Québec should increase transparency about restrictions on organ and tissue donation that affect some 2S/LGBTQI+ people, including by disclosing these restrictions to all potential donors and/or their medical decision maker.” (n = 1,602)	
Strongly agree	644 (40%)
Somewhat agree	346 (22%)
Neither agree nor disagree	275 (17%)
Somewhat disagree	87 (5.4%)
Strongly disagree	250 (16%)
Missing	470

2S/LGBTQI+: Two-Spirit, Lesbian, Gay, Bisexual, Transgender, Queer, Intersex, and more.

*Multiple responses were permitted; therefore, percentages do not sum to 100%.

## Discussion

This is the first published assessment of the self-reported predictive ability of behavior-based screening for organ and tissue donation via self- and secondary-report in 2S/LGBTQI+ communities. We demonstrated the perceived reliability of behavior-based donor screening varies substantially according to the relationship between the potential donor and substitute decision-maker. Those at highest risk for HIV reported lower confidence their substitute decisionmakers could accurately answer questions about their sexual partners. Approximately 1/5 survey participants reported they were uncertain or unwilling to disclose these behaviors to healthcare providers. More than half perceived the newly proposed Health Canada donor risk assessment criteria to remain discriminatory and required further change.

### Behavior-Based Screening

If behavioral screening is upheld, the relationship of the substitute decisionmaker must be considered, as partners were consistently reported to have greater knowledge of donors’ recent sexual behaviors than other family members. Given the very low residual HIV risk following contemporary NAAT, the incremental benefit of behavioral screening warrants critical re-evaluation, particularly as the national implementation of universal donor NAAT screening is underway. The residual risk of an undetected case of HIV in donated blood from all donors after a negative NAAT test is extremely low, with Canadian blood donor modelling estimating one potential undetected infection per 21.4 million donations.^28^ These estimates illustrate the substantial reduction in HIV transmission risk achieved with contemporary NAAT and provide important context when considering the incremental benefit of added behavior-based screening. Despite a negative NAAT test for HIV, HCV and HBV, in Canada, patients subsequently classified as increased infectious risk donors (IIRDs) based on behavioral or identity-related factors are excluded from tissue donation and cannot donate solid organs outside of an exceptional distribution process where the benefit to the recipient is felt to outweigh the risk of HIV or viral hepatitis transmission through transplantation.^19,21^ Patients are routinely advised by their transplant physicians to accept organ offers from IIRDs as data supports that they have significantly improved survival, quality of life and reduced healthcare costs compared with declining an IIRD offer to wait for a ‘baseline risk’ donor.^12,13^ Given this, we question whether the IIRD paradigm is helping transplant recipients, or causing them to harm themselves due to social stigmas upheld/reinforced by current OTDT healthcare practices. A review of IIRD donors in Alberta and Manitoba found that MSM as a risk factor was not associated with HIV or HCV but behavioral factors not currently a part of the IIRD paradigm were significantly associated with HCV (publication forthcoming). In Australia, behavioral risk factors reported by the next-of-kin of deceased organ donors were associated with HCV but were poorly predictive of HIV and HBV. Further, there were a significant number of infections occurring in donors with no disclosed risk factors, supporting a poor negative predictive value of behavioral screening.^29^

While behavior-based screening policies may be more equitable than identity-based screening, the application of policy to practice introduces the potential for stigma to lead to inequitable care. Caregivers of 2S/LGBTQI+ deceased donors have described sexual behaviors being assumed by the healthcare team based on a 2S/LGBTQI+ identity without any behavioral questions being asked.^6^ Concerningly, in a recent survey of Canadian organ donation healthcare workers only 75% reported always asking specifically about sexual behaviors regardless of the gender of the donor’s partner, with others only asking about sexual behaviors if the donor and partner were both men or assuming that two men in a relationship were sexually active.^8^ A majority of organ and tissue donation healthcare workers who have lived experience conducting donor risk assessments also felt sexual behavioral screening, especially when conducted with next-of-kin rather than self-report, was not reliable.^8^ Sexual behavioral screening risks harming transplant recipients leading to decisions (refusing an offer of an increased infectious risk organ) that are more likely to harm them. Such screening risks inequitable care for 2S/LGBTQI+ potential donors, and does not meaningfully reduce the risk of HIV, HCV or HBV transmission through transplantation when combined with existing testing practices (e.g., universal screening with NAAT). Based on risks, impacts, and lack of meaningful benefit to potential donors and recipients alike, we argue that the sexual behavior screening component of the donor risk assessment should be altogether removed in favor of universal NAAT screening in all donors.

### 2S/LGBTQI+ Response to New Screening Questions

Public health systems rarely have the opportunity to develop policy that is informed by such robust community feedback as the CBRC’s *Sex Now* survey offers. In its new list of behaviors associated with HIV, HCV and HBV, Health Canada has removed MSM as a criterion and introduced two new sexual behavior based items: 1) “persons who have had a new sexual partner in the preceding 3 months and have had anal sex in the preceding 3 months”, and 2) “persons who have had more than one sexual partner in the preceding 3 months and have had anal sex in the preceding 3 months”.^22^ A significant proportion of survey participants indicated they were unsure if they would answer accurately or would not donate in response to Health Canada’s new donor risk assessment questions. Survey participants reported they felt these questions needed to be further refined so more 2S/LGBTQI+ people could donate organs and tissues. Many said they lacked trust in the healthcare system and that additional efforts at reparation with 2S/LGBTQI+ communities, better transparency regarding system inequities, and improved training of healthcare staff in 2S/LGBTQI+ care were needed. Canadian Blood Services has recently launched professional training “2S/LGBTQI+ Inclusive Care for OTDT Healthcare Professionals” as an important step in addressing this gap.^30^

Health Canada continues to consider sex in the past 3 months with a person living with confirmed or suspected HIV to increase a donor’s risk of transmitting HIV, even with a negative NAAT test.^22^ Over half of participants felt sexual contact with people living with HIV who have undetectable viral levels should not be considered as increased risk for the HIV-negative partner. This community perspective is consistent with contemporary evidence and scientific consensus that shows HIV cannot be transmitted through sexual contact when the partner living with HIV has an undetectable viral level.^31-36^ Similarly, over half of participants said Health Canada should not consider recent use of HIV PrEP/PEP as increasing risk of HIV transmission through organ donation. PrEP is exceedingly effective at preventing HIV transmission through sexual contact (>99%)^31-36^ and transmission through non-medical injection drug use (>74%).^37-39^ Canadian and U.S. blood donation systems defer donations from people using PrEP due to a theoretical risk of a false negative HIV test.^40,41^ The actual risk of donor-derived HIV following recent PrEP/PEP use remains uncertain and warrants formal epidemiologic modelling to inform future policy. Despite this, any oral PrEP/PEP within the past 3 months and any injectable PrEP/PEP within the past 2 years were added as criterion that would increase a donor’s risk for transmitting HIV, meaning these donors can only donate solid organs through an exceptional distribution process and cannot donate tissues at all.^22^ Further epidemiologic residual risk assessments should be undertaken to establish the real risk of transmission of HIV in people using PrEP/PEP so that donation policy can be based in science rather than stigma.

### Strengths/Limitations

Strengths include a large community-derived sample of 2S/LGBTQI+ people living in Canada who were recruited for a general community health survey and not one exclusively about the topic of ODTD, increasing the general representativeness of the sample. This community sample collected detailed sociodemographics as well as self-reported sexual and substance related behaviors, providing important nuance unavailable in clinical datasets. Despite broad recruitment and inclusive criteria, our convenience sampling strategy limits extrapolation of our results to the entire 2S/LGBTQI+ population in Canada. Some racialized populations and those without formal post-secondary education were underrepresented in our sample. The electronic nature of the survey could have restricted participation from those with socioeconomic or digital/health literacy barriers. Non-response and social desirability bias may also have influenced patient responses despite anonymous administration.

### Future Directions

Updated epidemiologic risk assessment models are needed to understand the incremental benefit of potentially stigmatizing criteria on HIV PrEP/PEP use when added to other screening practices (e.g., universal NAAT testing). Health Canada should support a critical re-evaluation of the current increased infectious donor risk (IIDR) assessment criteria as they relate to HIV/HCV/HBV, including whether this label should be eliminated. The development and evaluation of public health campaigns in partnership with community which celebrate the valued contribution of 2SLGBTQIA+ OTDT donors could help destigmatize donation for the public and overcome mistrust of healthcare for the 2S/LGBTQI+ community, which are issues that have been perpetuated by historic OTDT policy and practices.^42^

## Conclusions

Sexual behavioral screening for donor risk assessments in 2S/LGBTQI+ people living in Canada is likely to be poorly predictive of the risk of HIV and viral hepatitis transmission through organ and tissue transplantation. Health Canada should support a re-evaluation of the current IIRD paradigm guided by evidence and patient/community values/preferences to maximize benefits and minimize harms to patients and to 2S/LGBTQI+ community members.

## Supplemental Material

Supplemental Material - Behavior-Based Screening in Organ and Tissue Donor Risk Assessments in Two-Spirit, Gay, Lesbian, Bisexual, Transgender, Queer, Intersex and Other Sexual and Gender Minority (2S/LGBTQI+) People: A Canada-wide Community Survey Supplemental Material for Behavior-Based Screening in Organ and Tissue Donor Risk Assessments in Two-Spirit, Gay, Lesbian, Bisexual, Transgender, Queer, Intersex and Other Sexual and Gender Minority (2S/LGBTQI+) People: A Canada-wide Community Survey by Murdoch Leeies, Kimia Rohani, David Collister, Karen Doucette, Emily Christie, Carmen Hrymak, Sonny Dhanani, Warren Fingrut, Tzu-Hao Lee, Ken Sutha, Cameron T. Whitley, Ben Klassen, Nathan Lachowsky in Canadian Journal of Kidney Health and Disease
