# Supplementary material for: Behavior-Based Screening in Organ and Tissue Donor Risk Assessments in Two-Spirit, Gay, Lesbian, Bisexual, Transgender, Queer, Intersex and Other Sexual and Gender Minority (2S/LGBTQI+) People: A Canada-wide Community Survey
Source: Can J Kidney Health Dis. 2026 Sep 30;13:20543581261474939. doi: 10.1177/20543581261474939 (PMC13769908; doi:10.1177/20543581261474939)
Supplement: Supplementary file 1 [file sj-pdf-1-cjk-10.1177_20543581261474939.pdf]

Sex Now 2025

Start of Block: Consent

Welcome to the Sex Now Survey!
Si vous préférez compléter le sondage en français, appuyez sur le bouton de langue en haut à droite.
Si prefiere responder la encuesta en español, presione el botón de idioma en la parte superior derecha.

Who's contributing to this project?
This survey is being led by the Community-Based Research Centre and the University of Victoria. The survey is called Sex Now and is for Two-Spirit, lesbian, gay, bisexual, trans, queer, and other people of diverse sexualities and genders (2S/LGBTQ+).

Our team includes 2S/LGBTQ+ community members and researchers, and is led by Dr. Nathan Lachowsky (a gay, white, cisgender man) who is a Professor at the University of Victoria and Research Director for the Community-Based Research Centre (nlachowsky@uvic.ca, 250-472-5739). The team also includes researchers at the University of British Columbia (Dr. Devon Greyson, Dr. Mark Gilbert, and Jeffrey Morgan), Simon Fraser University (Dr. Travis Salway, Dr. Kiffer Card, and Harlan Pruden), and and Université de Montréal (Dr. Olivier Ferlatte). We work closely with 2S/LGBTQ+ community leaders and organizations across the country.

The study is partially funded by grants from the Canadian Institutes of Health Research and Health Canada.

What is the survey about?
The purpose of the survey is to learn more about the health of Two-Spirit, lesbian, gay, bisexual, trans, queer and other people of diverse sexualities and genders (2S/LGBTQ+). The survey includes questions about sexually transmitted and blood borne infections (such as HIV, syphilis, and mpox), your sex life, mental health, access to health services, substance use, experiences of violence or discrimination, and connections to community. It also asks for your perspectives on blood, organ, and tissue donation policies for our communities and about your experiences with gambling. The experiences you share will allow us to better understand the services and supports most needed by 2S/LGBTQ+ people in Canada.

What will I be asked to do?
We will ask you to complete a confidential online survey on your computer, tablet, or phone. The online survey will take about **30 minutes** to complete. If you leave the survey to take a break

and return to it later using the same device and browser within two days, you will be able to continue from where you stopped previously.

Are there any risks involved in the survey?
We know that answering personal questions (such as those about substance use, discrimination, gambling, sexual abuse or barriers to accessing care or support) can be difficult and may upset or distress you. It is important to remember that most questions are optional and can be skipped at any time. If needed, here is a list of 2S/LGBTQ+ support resources which you can access. This includes a number of 24/7 crisis lines that can be reached at any time.

What are my rights?
This study is completely voluntary and your choice to participate will not impact your future access to any services. Your survey responses will be securely protected. If you choose to provide an email address at the end of the survey, this will be collected and stored separate from your survey responses and there will be no way for the research team to link your contact information and responses.

You don't have to answer any questions you don't want to answer, except for ones to ensure you are eligible to participate. You can also stop at any time and will not be penalized. If you stop before completing the survey, your responses up to that point will be kept by the research team. We will be unable to remove your survey responses after you participate because we will not know which responses belong to you. By consenting, you do not waive any rights to legal recourse in the event of research-related harm.

What are the benefits?
By participating, you are helping to generate data which will help to improve the health and well-being of 2S/LGBTQ+ people by providing researchers and community organizations with the information they need to make things better. You'll also get to access findings from the study online for free next year through the Community-Based Research Centre's website (www.cbrc.net).

Is there any compensation for participating?
If you complete the survey, you may choose to enter a draw for one (1) of three (3) five-hundred-dollar ($500.00) travel vouchers by entering your email address. If you are a winner, we will ask you to provide a name to allow us to send you the travel voucher.

How will survey data and emails be used?
Since data for this study is being collected online, there is a risk of a privacy breach where online data could be unintentionally accessed by someone outside of our research team. This includes on your personal device (e.g., your phone or computer), since you may be using applications that track your data. We are taking precautions to minimize this risk. The information you provide in the survey will be treated according to privacy laws including the Federal Personal Information Protection and Electronic Documents Act (PIPEDA). All survey data will be stored on encrypted Qualtrics servers during data collection, then stored on

University of Victoria servers in Canada that will only be accessed by people on our research team who have signed a confidentiality agreement and undergone privacy training. The Qualtrics license being used for this study is a personal license owned by Dr. Lachowsky. Any data you provide will be transmitted and stored in Canada.

If you provide an email address to join the CBRC mailing list, contact for future studies, and/or be entered into the prize draw, this information will be collected and stored separate from your survey responses and only be used for the purposes you consented to. Email addresses provided for the prize draw will be destroyed after winners have been contacted at the end of the study period. If you consent to be recontacted for future studies, we will keep your email address for five years. Additionally, we may contact you on behalf of approved researchers within our network for studies which we are not leading. We will not provide any of your personal information to these researchers. You may opt-out of the CBRC mailing list at any time by clicking unsubscribe in any communications from the organization. You may ask for removal from the future studies contact list by emailing sexnow@cbrc.net.

Who are you sharing data and findings with?
Your survey data may be shared with other researchers who are interested in the health and wellbeing of 2S/LGBTQ+ communities, including researchers outside of Canada. You will not be directly identifiable within this data. You may choose whether you agree to your survey data being used for future unspecified research or not. This will not impact your ability to participate in this study.

We will share our findings with academic, government, and community groups, including our funders. However, you will not be identified in these results. Researchers will at all times comply with the Tri-Council ethical guidelines for research with human participants. The information will be used to write reports, provide statistical information and to prepare presentations. You will not be identified in any way as these reports and other public documents will always refer to groups of people, never to one person.

Voluntary Participation
Participation in the study is completely voluntary. If you decide not to participate, it won't impact your eligibility for future studies or affect how you are treated by any health, treatment or social agencies. While completing the survey you can stop at any time (e.g., by closing your browser) and many questions can be skipped.

Who can I contact if I have any questions about the study?
If you have any questions or need further information with respect to this study, you may contact the research team at 604-568-7478 or at sexnow@cbrc.net. You can also review a copy of this consent form at any time by going to the Sex Now page of the CBRC website (https://www.cbrc.net/sex_now).

Who can I contact if I have a concern about my rights as a participant?

This research has been reviewed by the University of Victoria's Research Ethics Board and conforms to the standards of the Canadian Tri-Council Research Ethics guidelines. In addition, you may verify the ethical approval of this study, or raise any concerns you might have, by contacting the Human Research Ethics Office at the University of Victoria (250-472-4545 or ethics@uvic.ca).

Consent Agreement
Do you acknowledge and agree to the conditions outlined above?

○ Yes

○ No

Do you agree to your survey data being used for future unspecified research? This will not impact your ability to participate in this study.

○ Yes

○ No

End of Block: Consent

Start of Block: Demographics

Have you completed a Sex Now Survey before? Select all that apply.

- ☐ Yes, I have already completed the survey online in 2025
- ☐ Yes, in-person in 2024
- ☐ Yes, online in 2024
- ☐ Yes, in-person in 2022
- ☐ Yes, online in 2021
- ☐ Yes, online in 2020
- ☐ Yes, online in 2019
- ☐ Yes, in-person in 2018
- ☐ Yes, online in 2014/15
- ☐ ⊗ No, none of the above

Do you live in...?

- ◯ Alberta
- ◯ British Columbia
- ◯ Manitoba
- ◯ New Brunswick
- ◯ Newfoundland & Labrador
- ◯ Northwest Territories
- ◯ Nova Scotia
- ◯ Nunavut
- ◯ Ontario
- ◯ Prince Edward Island
- ◯ Quebec
- ◯ Saskatchewan
- ◯ Yukon
- ◯ I don’t live in Canada

How old are you? (In numerical years; for example enter “18” or “75” below)

____

Thank you for your interest in Sex Now 2025!
Based on your answers above, you don't seem to fit the eligibility criteria for this study, however, we greatly appreciate your time! If you have questions about eligibility, please ask a study team member by emailing sexnow@cbrc.net.

Our racial and ethnic identities may shape how we are treated by different individuals and institutions. The next questions ask about your race and ethnicity. We acknowledge these questions may result in uncomfortable feelings. We ask these questions to respond to the call by our community leaders and the Canadian Public Health Association for culturally safe collection and use of sociodemographic and race-based data. **How do you identify your race and ethnicity?**

____

Which of these do you identify with? Select all that apply.

- ☐ Black (e.g., Black African, Black Caribbean, Black Canadian)
- ☐ East Asian (e.g., Chinese, Korean, Japanese)
- ☐ Indigenous (e.g., First Nations, Métis, Inuk)
- ☐ Latina, Latino, Latinx, Latine (e.g., Mexican, Peruvian, Brazilian)
- ☐ Southwest Asian and North Africa (e.g. Afghan, Egyptian, Moroccan)
- ☐ South Asian (e.g., East Indian, Pakistani, Bangladeshi, Sri Lankan)
- ☐ Southeast Asian (e.g., Cambodian, Indonesian, Vietnamese)
- ☐ White (e.g., European descent)
- ☐ ⊗ None of the above

We use the term “racialized persons” to acknowledge the social impact of racism and racialization in Canada. Sometimes people use terms like “people of colour” or “BIPOC” or “visible minorities” to mean the same thing. **Do you identify as a racialized person?**

- ○ Yes
- ○ No
- ○ I'm not sure

Do other people in Canada usually see or treat you as a person of colour / racialized person?

- ○ Yes
- ○ No
- ○ Unsure

Are you Two-Spirit?

- ○ I am Two-Spirit
- ○ I am not Two-Spirit
- ○ I prefer not to answer this question

As an Indigenous person, do you identify as…? Select all that apply.

- ☐ First Nations
- ☐ Métis
- ☐ Inuk
- ☐ Indigenous from somewhere else in the world (e.g. Torres Strait)
- ☐ Other, please specify: ______________________________

Which term(s) do you use to describe your sexual orientation? Select all that apply. The options are listed in alphabetical order.

- ☐ Asexual
- ☐ Bisexual
- ☐ Demisexual
- ☐ Gay
- ☐ Heteroflexible
- ☐ Homoflexible
- ☐ Lesbian
- ☐ Pansexual
- ☐ Queer
- ☐ Questioning
- ☐ Straight
- ☐ I prefer to use another term (please specify): ______________________

The next four questions are about gender and sex. We recognize that identities and labels are important parts of our lives and how we think about ourselves. We also recognize that people use different terms to define their sex and gender. We ask these questions to compare with other data collected nationally and globally. We apologize if these response options may not be a perfect fit for you.

Do you identify as trans?

- ○ Yes
- ○ No
- ○ Unsure or questioning

What is your gender identity? Select all that apply. If you have lived experience as trans, a history of gender transition, or are transgender, please select the gender you identify as. We recognize that gender identity questions are imperfect. **Please select the options that fit best at this time.** The options are listed in alphabetical order.

- ☐ Agender
- ☐ Genderfluid
- ☐ Genderqueer
- ☐ Man
- ☐ Non-binary
- ☐ Questioning
- ☐ Trans man
- ☐ Trans woman
- ☐ Transfeminine
- ☐ Transmasculine
- ☐ Woman
- ☐ I prefer to use another term: ______________________________

What was your sex assigned at birth? We acknowledge that questions about one's assigned sex may result in uncomfortable feelings/memories. We are asking this question so that we can use the information we get from this survey to better inform services for trans people.

- ○ Male
- ○ Female
- ○ I prefer not to answer

Are you an intersex person? Intersex is a term for people born with a variation of sex characteristics. There are many different intersex traits or variations.

- ○ Yes
- ○ No
- ○ Unsure

In the PAST 12 MONTHS, who did you have sex with in terms of gender? We define sex as any physical contact that you felt was sexual. Select all that apply.

- ☐ A cis man
- ☐ A cis woman
- ☐ A trans man
- ☐ A trans woman
- ☐ A non-binary person
- ☐ An agender person
- ☐ A genderfluid person
- ☐ A genderqueer person
- ☐ ⊗ None of the above

Thank you for your interest in Sex Now 2025!
Based on your answers above, you don't seem to fit the eligibility criteria for this study, however, we greatly appreciate your time! If you have questions about eligibility, please ask a study team member by emailing sexnow@cbrc.net.

On a scale from 1 to 5, how open or out are you about your... Note: outness is complicated and we affirm everyone's right to navigate information they share (or don't) about their identities.

	1-Not at all open	2	3	4	5-Open (out) to all or most people
**Gender Identity**	☐	☐	☐	☐	☐
**Sexual Identity**	☐	☐	☐	☐	☐

Are you currently in a relationship?

- ◯ Yes
- ◯ No

How many people are you in a relationship with? Enter 0 if you do not have any partners of that gender.

- ◯ Men ______________________________
- ◯ Women ______________________________
- ◯ Non-binary people ______________________________
- ◯ Other ______________________________

Do you identify as a person with a disability?

- ◯ Yes
- ◯ No
- ◯ Unsure

Would you share the first three characters of your postal code? We ask this question to locate your response to a region, not your specific address. Different regions of Canada have different health policies, services, and programs.

- ◯ Yes
- ◯ I don’t know my postal code
- ◯ I do not have a postal code
- ◯ I do not want to provide this information

What are the first three characters of your postal code? Your response should be in this format: Letter-Number-Letter (e.g. V2N, M3N, L4M).

____

What best describes the environment you live in?

- ○ Very large urban population centre (500,000+ people)
- ○ Large urban population centre (100,000-499,999 people)
- ○ Medium population centre (30,000-99,999 people)
- ○ Small population centre (1,000-29,999 people)
- ○ Rural area (Less than 1,000 people)

Do you currently live on a First Nations reserve?

- ○ Yes
- ○ No

Were you born in Canada?

- ○ Yes
- ○ No

In what year did you first move to Canada? Please enter a four-digit year (e.g., 2015).

____

What is your current immigration status? Select all that apply. We acknowledge that questions around immigration status often result in barriers to access or denial of services, particularly for those with precarious immigration statuses. Your responses to this survey will remain confidential. At no time will this information be shared with Canada Border Service Agency (CBSA), or any government agencies that enforce immigration policy.

- ☐ A Canadian citizen
- ☐ A permanent resident (landed immigrant)
- ☐ A person without status (undocumented person, irregular migrant)
- ☐ A refugee or asylum seeker
- ☐ A temporary foreign worker (work permit)
- ☐ A visitor
- ☐ An international student (study permit)
- ☐ Other, please specify: ______________________
- ☐ ⊗ None of the above

Why did you move to Canada? Select all that apply.

- ☐ For better health and social services
- ☐ For connections with family / friends
- ☐ For economic or job opportunities
- ☐ For education
- ☐ For romantic reasons
- ☐ For the stronger visibility of 2S/LGBTQIA+ communities
- ☐ To acquire legal recognition of relationship (e.g. marriage) and/or my gender (e.g., name, gender marker on ID)
- ☐ To avoid discrimination based on racial and/or ethnic minority status
- ☐ To avoid discrimination based on religion and/or politics
- ☐ To avoid discrimination based on sexual and/or gender minority status
- ☐ To avoid war-related violence
- ☐ To flee from persecution (e.g., violence, death threat, imprisonment) based on sexual and/or gender minority status
- ☐ Other, please specify: ______________________________
- ☐ ⊗ None of the above

How would you describe your English and French language skills? (Please choose the best answer for each language)

	Limited/None	Beginner	Intermediate	Advanced	Native
**English**	☐	☐	☐	☐	☐
**French**	☐	☐	☐	☐	☐

There have been many changes to immigration policies in Canada in the past few years, such as reducing the number of approved international students, foreign workers, and refugees. **Have immigration policy changes affected you in any of the following ways in the PAST THREE YEARS?** Select all that apply.

- ☐ Made it harder for me to get my student permit in Canada
- ☐ Made it harder for me to get my work permit in Canada
- ☐ Made it harder for me to get my refugee status in Canada
- ☐ Made it harder for me to get my permanent residency in Canada
- ☐ Made it harder to sponsor my family member(s) to come to Canada
- ☐ Negatively affected my mental health
- ☐ Negatively affected my physical health
- ☐ Made me feel hopeless
- ☐ Other, please specify: ____________________
- ☐ ⊗ None of the above

Overall, how would you describe your money situation in the PAST 6 MONTHS?

- ○ Comfortable, with extra
- ○ Enough, but no extra
- ○ Have to cut back
- ○ Cannot make ends meet

In the PAST 6 MONTHS, what types of places have you lived in? Select all that apply.

- ☐ In my apartment, condo, or house
- ☐ In another person's apartment, condo, or house
- ☐ Student residence/housing
- ☐ Hotel or motel room
- ☐ Rooming or boarding house
- ☐ Shelter or hostel
- ☐ Transition house or halfway house
- ☐ Psychiatric institution
- ☐ Treatment facility, like detox or rehab
- ☐ Public place, like a street, park or stairwell
- ☐ In a vehicle (e.g., car, van, RV, truck)
- ☐ ⊗ None of the above

Have you EVER been homeless, meaning having to live in a homeless shelter, on the street or in parks, in a makeshift shelter or in an abandoned building? Select all that apply.

- [ ] Yes, in the past 6 months
- [ ] Yes, but more than 6 months ago
- [ ] ⊗ No, never

Have you EVER spent time in a correctional facility? For example, a jail or prison. Select all that apply.

- [ ] Yes, in the past 6 months
- [ ] Yes, but more than 6 months ago
- [ ] ⊗ No, never

What has been your employment status during the PAST 6 MONTHS? Select all that apply.

- ☐ Employed full-time (30+ hours/week)
- ☐ Employed part-time (less than 30 hours/week)
- ☐ Self-employed: professional, contractor, business owner
- ☐ On government assistance
- ☐ Student
- ☐ Retired
- ☐ On leave (e.g., parental, caregiving)
- ☐ Unemployed
- ☐ Unable to work
- ☐ ⨂None of the above

What is the highest level of education that you have completed?

- ○ Did not finish high school
- ○ High school or equivalent
- ○ Post-secondary school (e.g. certificate, diploma, CEGEP)
- ○ Bachelor's degree
- ○ Above a bachelor's degree (e.g. masters, doctorate)

End of Block: Demographics

Start of Block: Knowledge

The questions in this section are about your knowledge concerning HIV and STI prevention and treatment. **Please read the following statements. For each, select if you knew this before taking this survey or not.**

	Yes, I knew this already	No, I did not know this
HIV Pre-Exposure Prophylaxis ("PrEP") is HIV medication that HIV-negative people can take before and continuing after sex to prevent getting HIV.	▢	▢
There is a new form of <u>HIV PrEP</u> that involves receiving an injection in your butt cheek every 2 months to prevent HIV. This is called injectable HIV PrEP.	▢	▢
HIV Post-Exposure Prophylaxis ("PEP"): Within 3 days after a sexual risk event an HIV-negative person takes HIV medication for a month that may stop HIV from taking hold in the body.	▢	▢
HIV medications, taken consistently by someone living with HIV, can make their HIV viral load undetectable.	▢	▢
There is a new form of <u>HIV treatment</u> that involves receiving two injections in your butt cheek every 2 months. This is called injectable HIV treatment.	▢	▢
There is now scientific consensus that someone who remains HIV viral load undetectable cannot pass HIV to their sexual partners. This is known as "Undetectable = Untransmittable" (U=U).	▢	▢
There is a new way to reduce the chance of getting STIs like syphilis and chlamydia by taking an antibiotic called doxycycline. This is called Doxy PrEP (taken before sex) or Doxy PEP (taken after sex). This does not protect against HIV.	▢	▢
Syphilis can be passed from giving and receiving oral sex, including rimming.	▢	▢
Syphilis can be passed via vagina/front hole or anal sex.	▢	▢
There is an effective vaccine for Human Papilloma Virus that reduces your risk of oral, anal, cervical, penile and throat cancers, and reduces your risk of genital and anal warts.	▢	▢
In Canada, the number of new chlamydia and gonorrhea diagnoses are increasing.	▢	▢

End of Block: Knowledge

Start of Block: Organ and Tissue Donation and Transplantation

In Canada, we do not have enough human organs (e.g., kidneys, lungs, heart, liver) or tissues (e.g. corneas, blood vessels, bone) available for transplantation in people who need them.

In this section we hope to understand your knowledge and opinions about current organ and tissue donation and transplantation policies in Canada as well as your thoughts on how we could improve this system.

The next questions are about what you know about current organ and tissue donation policies. **Please read the following statements. For each, select if you knew this before taking this survey or not.**

	Yes, I knew this already	No, I did not know this
Prior to March 2025, Most trans and non-binary people who wanted to donate ORGANS or TISSUES were screened based on their sex-assigned-at-birth rather than their self-identified gender.	☐	☐
Prior to March 2025, MenHealth Canada policies consider “men” to be people who are assigned male at birth regardless of current gender identity who had any kind of sex with other men within the PAST 12 MONTHS were considered to have an INCREASED RISK of transmitting HIV or hepatitis through ORGAN DONATION. They could still donate organs (e.g., kidney), but the person receiving an organ was told it was “increased risk.”	☐	☐
Prior to March 2025, MenHealth Canada policies consider “men” to be people who are assigned male at birth regardless of current gender identity who had any kind of sex with other men within the PAST 12 MONTHS were considered to have an INCREASED RISK of transmitting HIV or hepatitis through TISSUE DONATION. They could not donate tissues at all.	☐	☐
In March 2025, the Canadian Standards Association proposed removing policies for “men who have sex with men” and proposed asking gender-neutral questions of all potential organ and tissue donors: “Have you had any new sexual partners or more than one sexual partner in the last three months?” If yes, “have you had anal sex in the past 3 months?” If yes, you would be considered INCREASED RISK in ORGAN donation and could not donate tissues.	☐	☐
In March 2025, the Canadian Standards Association proposed reducing the period where a potential donor is considered “increased risk” from 12 months to 3 months.	☐	☐
In March 2025, the Canadian Standards Association proposed a policy where any sexual contact with a person living with HIV in the past 3 months (a reduction from 12 months), even if they are undetectable and cannot pass HIV through sex, is considered INCREASED RISK in ORGAN donation.	☐	☐

In March 2025, the Canadian Standards Association introduced NEW proposed criteria - that people who have taken oral HIV PrEP or PEP in the past 3 months or injectable HIV PrEP in the past 2 years should be considered INCREASED RISK in ORGAN donation (and not allowed to donate tissues at all while this applies).	☐	☐

Policy Alternatives & Change

Health Canada's proposed new organ and tissue donation policies include shifting to gender-neutral sexual behaviour questions instead of asking questions specifically about "men who have sex with men."

Would you answer these proposed gender-neutral questions if asked by the transplant team…

	Yes	No (I would no longer donate)	Unsure
"Have you had sex with a new partner in the last 3 months?"	☐	☐	☐
"Have you had sex with more than 1 partner in the last 3 months?"	☐	☐	☐
"Have you had any anal sex in the last 3 months?"	☐	☐	☐
"Have you used oral HIV PrEP/PEP in the last 3 months or injectable HIV PrEP in the last 2 years?"	☐	☐	☐

Please rate your level of agreement with the statements below. Consider the new Health Canada policy (proposed in March 2025) outlined in the previous questions when responding.

	Strongly agree	Somewhat agree	Neither agree nor disagree	Somewhat disagree	Strongly disagree
The proposed Health Canada organ and tissue donation policy is **justified**	☐	☐	☐	☐	☐
The proposed Health Canada organ and tissue donation policy is **based on the best available research evidence**	☐	☐	☐	☐	☐
The proposed Health Canada organ and tissue donation policy is **discriminatory**	☐	☐	☐	☐	☐
The proposed Health Canada organ and tissue donation policy **requires further change**	☐	☐	☐	☐	☐
Health Canada should not consider sexual contact with people living with HIV who are undetectable as increasing risk for the HIV-negative partner	☐	☐	☐	☐	☐
Health Canada should not consider recent use of HIV PEP or PrEP as increased risk in organ donation	☐	☐	☐	☐	☐

The next question is about your willingness to donate organs or tissues. **If you were eligible, which of the following would you be willing to donate?** Select all that apply.

- ☐ ORGANS after my death (e.g., kidneys, liver, lungs, heart, pancreas, small bowel)
- ☐ TISSUES after my death (e.g., corneas from my eyes, blood vessels, bone)
- ☐ ORGANS (e.g., kidney or a part of my liver) as a LIVING organ donor
- ☐ STEM CELLS as a LIVING donor
- ☐ ⊗ None of the above

All organ and tissue donors are tested for HIV and hepatitis prior to donation. If a potential donor has very recently gotten HIV or hepatitis there is a chance that this test could be inaccurate and they could pass HIV or hepatitis through transplantation.

To reduce this risk, all potential donors are asked about behaviors, including some sexual and substance use practices associated with getting HIV or hepatitis. These questions are asked directly to potential living donors or more commonly to their next of kin (e.g., partner or family) for people donating after death.

The following are some questions currently used to screen potential organ and tissue donors for "increased risk" of HIV and hepatitis. Please answer the following questions to the best of your ability. We will ask you later about your willingness to respond to these questions.

Have you had sex in exchange for money or drugs in the LAST 3 MONTHS?

- Yes
- Maybe
- No
- I prefer not to answer

Have you had sex in the LAST 3 MONTHS with a person who had sex in exchange for money or drugs in the LAST 3 MONTHS?

- Yes
- Maybe
- No
- I prefer not to answer

Have you had sex in the LAST 3 MONTHS with a person who used non-medical injection drugs in the LAST 3 MONTHS?

- ◯ Yes
- ◯ Maybe
- ◯ No
- ◯ I prefer not to answer

The next questions are related to organ and tissue donation that would occur after your death.

Who is your current medical decision maker? This is a person who makes decisions about your medical care if you are unable to do so yourself and would normally be your next of kin unless you have specifically chosen someone as your medical decision maker.

- ◯ My partner
- ◯ My child
- ◯ My parent
- ◯ My sibling
- ◯ A member of my extended family
- ◯ Someone else I have chosen as my medical decision maker (e.g. friend)
- ◯ I don’t know

How likely is it that your medical decision maker would accurately answer questions about…?

	Very likely	Somewhat likely	Neither likely nor unlikely	Somewhat unlikely	Very unlikely
**Your recent sex life** (e.g., whether you had sex in exchange for money or drugs)	☐	☐	☐	☐	☐
**Details about your recent sexual partner(s)** (e.g., their HIV status, their use of injection drugs, whether they had sex in exchange for money or drugs)	☐	☐	☐	☐	☐
**Your recent substance use** (e.g., cocaine, non-medical injection drug use)	☐	☐	☐	☐	☐
**Your recent use of HIV PrEP or PEP**	☐	☐	☐	☐	☐

The next questions are about organ donation as a living donor (e.g., giving one of your kidneys or part of your liver). As a reminder, living organ donors who are considered "increased risk" can still donate organs (e.g., kidney), but the person receiving an organ will be told it is "increased risk."

Would you answer questions asked by the transplant team about…

	Yes	No (I would no longer donate)	Unsure
**Your recent sex life** (e.g., whether you had sex in exchange for money or drugs)	☐	☐	☐
**Details about your recent sexual partner(s)** (e.g., their HIV status, their use of injection drugs, whether they had sex in exchange for money or drugs)	☐	☐	☐
**Your recent substance use** (e.g., cocaine, non-medical injection drug use)	☐	☐	☐
**Your recent use of HIV PrEP or PEP**	☐	☐	☐

Do you trust the health system and providers enough to provide accurate responses to the previous set of questions?

- ○ Yes
- ○ No
- ○ Unsure

What additional organ and tissue donation changes would you like to see? Select all that apply.

- ☐ More precise screening for sexual practices to allow more 2S/LGBTQIA+ people to donate
- ☐ Improved sexual and gender diversity training for donation staff
- ☐ 2S/LGBTQIA+-specific outreach efforts (e.g., relationship building, repair work, targeted recruitment efforts)
- ☐ Promotion of donation options by 2S/LGBTQIA+ community organizations
- ☐ Other. Please specify: ____________________
- ☐ ⊗ None of the above

Do you believe that Health Canada, Canadian Blood Services and Transplant Québec should apologize and make reparations to the 2S/LGBTQIA+ community for existing organ and tissue donation policies?

- ○ Yes
- ○ No
- ○ I'm not sure

Please rate your level of agreement with the following statement. "Health Canada, Canadian Blood Services and Transplant Québec should increase transparency about restrictions on organ and tissue donation that affect some 2S/LGBTQIA+ people, including by disclosing these restrictions to all potential donors and/or their medical decision maker."

- Strongly disagree
- Somewhat disagree
- Neither agree nor disagree
- Somewhat agree
- Strongly agree

End of Block: Organ and Tissue Donation and Transplantation

Start of Block: Sex life

The questions in this section are about your sex life and relationships. We define sex as any physical contact that you felt was sexual.

The next question is about your experience with "new sex partners". This means someone you had never had sex with before. **When did you last have a "new sex partner"?**

- This week
- 2-4 weeks ago
- 1-3 months ago
- 4-6 months ago
- 7-12 months ago
- Longer than a year ago
- I have never had sex

In the PAST 12 MONTHS, where did you look for sex or meet sexual partners? Select all that apply.

- ☐ Online (apps, websites, chat rooms)
- ☐ Social venues (includes community organizations, recreational groups, 2S/LGBTQIA+ community events, pop-up queer events/parties)
- ☐ Bars (includes gay and straight bars, and after-hours clubs/parties)
- ☐ Through friends and/or existing partners
- ☐ Saunas or bathhouses (baths)
- ☐ Rave/circuit parties or other commercial sex parties
- ☐ Private sex parties in someone's home
- ☐ Public settings (includes bicycle paths, parks, public restrooms, stairwells, health clubs/gyms)
- ☐ Other
- ☐ ⊗ None of the above

How many sex partners have you had in the PAST 12 MONTHS?

____

We ask the next question so that we can ask you about sexual activities that are relevant to you. We all have different ways we talk about our bodies, and different words to refer to our body parts. We apologize if these are not the terms you use. Note that for this question, we are not including prosthetics, strap-ons, or sex toys.

Did you have sex using these parts of your body in the PAST 12 MONTHS? Select all that apply.

- [ ] My bum
- [ ] My vagina or front hole
- [ ] My mouth
- [ ] My penis
- [ ] ⊗ None of the above

In the PAST 12 MONTHS, what kinds of sex have you had? Select all that apply.

- [ ] Masturbating or fingering someone
- [ ] Someone masturbating or fingering you
- [ ] Giving oral sex (your mouth on someone else's genitals)
- [ ] Receiving oral sex (someone else's mouth on your genitals)
- [ ] Rimming (your mouth on someone else's butt)
- [ ] Getting rimmed (someone else's mouth on your butt)
- [ ] Fisting someone else’s vagina/front hole
- [ ] Getting my vagina/front hole fisted
- [ ] Fisting someone else’s butt
- [ ] Getting my butt fisted
- [ ] Scissoring or frotting (rubbing your genitals on someone else’s genitals without penetration)

- [ ] Vaginal/front hole sex as top/insertive partner (your penis in someone else's vagina/front hole)
- [ ] Vaginal/front hole sex as bottom/receptive partner (someone else's penis in your vagina/front hole)
- [ ] Anal sex as top/insertive partner (your penis in someone else's butt)
- [ ] Anal sex as bottom/receptive partner (someone else's penis in your butt)
- [ ] Threesome (sex with 2 other people)
- [ ] Group sex (sex with 3 or more other people)
- [ ] ⊗ None of the above

In the PAST 12 MONTHS, what kinds of sex have you had? Select all that apply.

- [ ] Sex with prosthetics, strap-ons, or sex toys
- [ ] BDSM, kink, or fetish play
- [ ] Online or phone sex (camming, sexting)
- [ ] ⊗ None of the above

In the PAST 12 MONTHS, did you EVER NOT use a condom/barrier (e.g., dental dam for oral sex, glove for fisting) when you had these types of sex? Select all that apply.

- ☐ Masturbating or fingering someone
- ☐ Someone masturbating or fingering you
- ☐ Giving oral sex (your mouth on someone else's genitals)
- ☐ Receiving oral sex (someone else's mouth on your genitals)
- ☐ Rimming (your mouth on someone else's butt)
- ☐ Getting rimmed (someone else's mouth on your butt)
- ☐ Fisting someone else’s vagina/front hole
- ☐ Getting my vagina/front hole fisted
- ☐ Fisting someone else’s butt
- ☐ Getting my butt fisted
- ☐ Vaginal/front hole sex as top/insertive partner (your penis in someone else’s vagina/front hole)
- ☐ Vaginal/front hole sex as bottom/receptive partner (someone else’s penis in your vagina/front hole)
- ☐ Anal sex as top/insertive partner (your penis in someone else’s butt)
- ☐ Anal sex as bottom/receptive partner (someone else’s penis in your butt)
- ☐ Sex with prosthetics, strap-ons, or sex toys
- ☐ Threesome (sex with 2 other people)
- ☐ Group sex (sex with 3 or more other people)

☐ ⊗ None, I used condoms/barriers for all types of sex I had in the PAST 12 MONTHS

In the PAST 12 MONTHS, who did you have sex with? Select all that apply.

☐ A person who was on HIV PrEP

☐ A person who was HIV undetectable

☐ A person who I believed to be HIV-negative

☐ A person who I believed to be HIV-positive

☐ A person whose HIV status I did not know

☐ A recurring sex partner (someone you've had sex with more than once)

☐ A one-off sex partner (someone you've had sex with only once)

☐ A person who gave me money, goods or services for sex

☐ A person who I gave money, goods or services for sex

☐ ⊗ None of the above

End of Block: Sex life

Start of Block: STIs

The questions in this section are about sexually transmitted infections (STIs) other than HIV, such as syphilis, chlamydia, or gonorrhea.

When were you LAST tested for any STIs (other than HIV)?

- ◯ In the past 3 months
- ◯ 4-6 months ago
- ◯ 7-12 months ago
- ◯ Longer than a year ago
- ◯ I have never tested for STIs (other than HIV)
- ◯ I don't know

What type of STI tests have you received in the PAST 12 MONTHS (other than HIV)?
Select all that apply.

- ☐ Urine test
- ☐ Blood sample (usually from arm)
- ☐ Throat swab
- ☐ Rectal swab (in your bum)
- ☐ Vaginal (front hole) or cervical swab
- ☐ Urethral swab (in your penis)
- ☐ A pelvic exam
- ☐ Finger prick blood sample
- ☐ I don't know
- ☐ ⊗ None of the above

Have you had any of the following infections that can be transmitted sexually? If so, when was the last time? The options are listed in alphabetical order.

	Yes, in the past 12 months	Yes, more than 12 months ago	No, never
Bacterial vaginosis	☐	☐	☐
Chlamydia	☐	☐	☐
Crabs (pubic lice)	☐	☐	☐
Gonorrhea	☐	☐	☐
Hepatitis C virus	☐	☐	☐
Herpes (genital or anal)	☐	☐	☐
Lymphogranuloma venereum (LGV)	☐	☐	☐
Mpox (formerly known as monkeypox)	☐	☐	☐
Non-specific urethritis	☐	☐	☐
Shigella	☐	☐	☐
Syphilis	☐	☐	☐
Trichomoniasis	☐	☐	☐
Genital or anal warts	☐	☐	☐
Other (whether you know the name or not)	☐	☐	☐

Have any of the following caused you to delay or skip STI testing in the PAST 12 MONTHS? Select all that apply.

- ☐ Reduced (or no) sexual partners
- ☐ Too busy
- ☐ Services too far away
- ☐ Hours inconvenient
- ☐ Lack of privacy
- ☐ Lack of professional sensitivity to sexual and gender diversity
- ☐ Negative reaction from/judgement by health care worker
- ☐ Stressed out, anxious, or depressed
- ☐ The cost (e.g., no health insurance)
- ☐ Wait time for appointment too long
- ☐ Didn't know where to go
- ☐ Services not in my preferred language
- ☐ I’m in a closed sexual relationship
- ☐ Other
- ☐ ⊗ I did not delay or skip STI testing in the past year

Have you EVER been offered testing for HIV and/or STIs by a regular family doctor or nurse practitioner?

- ◯ Yes
- ◯ No
- ◯ I don’t know

Have you EVER taken Doxy PrEP/Doxy PEP to reduce the risk of getting STIs like syphilis or chlamydia? **This is not the same as HIV PrEP/PEP.**

- ◯ Yes
- ◯ No
- ◯ I don't know

When did you last take Doxy PEP (taken after sex)?

- ◯ In the past 6 months
- ◯ 7-12 months ago
- ◯ Longer than 12 months ago
- ◯ Never

When did you last take Doxy PrEP (taken before sex)?

- ◯ In the past 6 months
- ◯ 7-12 months ago
- ◯ Longer than 12 months ago
- ◯ Never

Are you interested in taking Doxy PrEP/PEP ?

- ◯ Yes
- ◯ No
- ◯ Unsure

Do you have or have you had a cervix?

- ◯ Yes
- ◯ No

In the LAST 3 YEARS, have you had a pap test or pap smear?

- ◯ Yes
- ◯ No
- ◯ Unsure
- ◯ Not applicable

What needs to be done to address STIs and HIV in 2S/LGBTQIA+ communities? Please share your recommendations and priorities.

____

____

____

____

End of Block: STIs

Start of Block: HIV

The questions in this section are about HIV.

Have you EVER been diagnosed with HIV? This is an important question. It determines whether you will be asked questions about living with HIV or questions about HIV testing and HIV pre-exposure prophylaxis (PrEP). We use this information to improve services and access. We keep this information confidential and only use it for research purposes.

- ○ Yes (I am living with HIV)
- ○ No (I have never been diagnosed with HIV)
- ○ Prefer not to answer

In the PAST 12 MONTHS have you done any of the following to reduce your chances of getting or passing on HIV? Select all that apply. These are different ways that you tried to reduce passing HIV through sex.

- ☐ Took HIV medications to be "undetectable" (low HIV viral load)
- ☐ Took Post-Exposure Prophylaxis (PEP) after sex where I might have gotten HIV
- ☐ Took Pre-Exposure Prophylaxis (PrEP) before and after sex where I might get HIV
- ☐ Only have condomless sex if a partner was "undetectable" (low HIV viral load)
- ☐ Only had condomless sex if a partner had the same HIV status as me
- ☐ Only had condomless sex if a partner was on PrEP
- ☐ Always used condoms during sex
- ☐ Had sex that didn't include anal or vaginal (front hole) sex
- ☐ Asked my sex partners about their HIV status before sex
- ☐ Only had sex in a closed/monogamous relationship
- ☐ Reduced my number of sexual partners
- ☐ Bottoming if you're living with HIV, topping if you're HIV negative

☐ ⊗ I have not used any of the HIV prevention practices above in the past 12 months

When were you LAST tested for HIV?

- ○ In the past 3 months
- ○ 4-6 months ago
- ○ 7-12 months ago
- ○ Longer than a year ago
- ○ I have never been tested for HIV
- ○ I don't know

What year did you first test positive for HIV? Please enter a 4-digit number.

____

Are you currently taking medications to treat HIV (i.e. antiretroviral drugs)?

- ○ Yes, I am currently taking antiretroviral drugs
- ○ No, but I have previously taken antiretroviral drugs
- ○ No, I have never taken antiretroviral drugs

How are you taking medications to treat HIV (i.e. antiretroviral drugs)?

- ○ Daily oral pill antiretroviral drugs
- ○ Long-acting injectable antiretroviral drugs
- ○ Other, please specify: ________________________________

What was the result of your most recent viral load test?

- ○ Suppressed (“undetectable” or less than 200 copies/ml)
- ○ Not suppressed (greater than 200 copies/ml)
- ○ I do not know or I am not sure
- ○ I have never had an HIV viral load test

The next 3 questions are about an injectable **LONG-ACTING HIV treatment** that is currently available. The medication is a combination of two drugs, cabotegravir and rilpivirine, and you would receive two injections (one in each butt cheek) every 2 months.

For you, what would be the benefits of an injectable long-acting HIV medication (taken every 2 months)? Select all that apply.

- ☐ Not needing to take a daily pill
- ☐ Not having HIV medication in my home
- ☐ Not having to store my HIV medication
- ☐ Not having to think about HIV every day
- ☐ Not worrying about forgetting to take my meds daily
- ☐ More frequent visits to my healthcare provider
- ☐ Increased mental well-being
- ☐ Increased social well-being
- ☐ Reduced stigma
- ☐ Reduced fear of violence from possible HIV disclosure
- ☐ More freedom to live my life

- [ ] Other, please specify:
____
- [ ] ⊗ None of the above

For you, what would be the drawbacks of an injectable long-acting HIV medication (taken every 2 months)? Select all that apply.

- [ ] Pain from injection
- [ ] Scared of needles
- [ ] More frequent visits to my healthcare provider
- [ ] Concern about cosmetic side effects from receiving an injection
- [ ] Concern about other side effects
- [ ] Concern it might not work as well as pills
- [ ] Worry about the cost
- [ ] Concern about whether it will suppress my viral load
- [ ] Decreased mental well-being
- [ ] Decreased social well-being
- [ ] Increased pressure to maintain an undetectable viral load
- [ ] Increased stigma
- [ ] Other, please specify:
____
- [ ] ⊗ None of the above

If both were available, what form of HIV treatment would you prefer?

- ◯ I would prefer daily oral pills
- ◯ I would prefer injections of long-acting medication every two months
- ◯ I don’t have a preference
- ◯ Unsure

End of Block: HIV

Start of Block: HIV PrEP

The questions in this section are about HIV Pre-Exposure Prophylaxis (PrEP).

Have you EVER taken HIV PrEP?

- ◯ Yes, I am currently taking HIV PrEP
- ◯ Yes, but I stopped in the past 6 months
- ◯ Yes, but I stopped more than 6 months ago
- ◯ No

How are you using HIV PrEP?

- ◯ Daily oral pills
- ◯ On-demand oral pills
- ◯ Other, please specify: ____

Have you EVER tried to get HIV PrEP?

- ◯ Yes
- ◯ No

Are you interested in using HIV PrEP?

- ◯ Yes
- ◯ No
- ◯ Unsure

What stops you from taking HIV PrEP? Select all that apply.

- ☐ I don't think I'll get HIV
- ☐ It costs too much (e.g., need to pay out of pocket, insurance doesn't cover PrEP)
- ☐ I can't get a prescription (e.g., don't know where, don't have a healthcare provider, not eligible)
- ☐ I'm worried about side effects (long- or short-term)
- ☐ I don't want to take a medication every day
- ☐ Too much testing and clinic visits
- ☐ I'm worried about judgement from people in my community
- ☐ I'm worried about judgement by a healthcare provider
- ☐ It doesn't provide protection from other STIs
- ☐ I don't think PrEP will fully protect me from HIV
- ☐ I would rather use condoms
- ☐ I need more information
- ☐ I'm worried that HIV medications would not work if I got HIV
- ☐ Reduced (or no) sexual partners

- [ ] I'm in a monogamous/closed relationship
- [ ] I wouldn't want others to know I am on HIV PrEP
- [ ] ⊗ None of the above

The next 3 questions are about an injectable **LONG-ACTING HIV PrEP** that was recently approved by Health Canada. The medication is called cabotegravir and involves receiving one injection in the butt cheek every 2 months.

For you, what would be the benefits of an injectable long-acting HIV PrEP (taken every 2 months)? Select all that apply.

- [ ] Not needing to take a daily pill
- [ ] Not having HIV PrEP medication in my home
- [ ] Not having to store my HIV PrEP medication
- [ ] Not having to think about HIV every day
- [ ] No worry about forgetting to take my oral HIV PrEP daily
- [ ] More frequent visits to my healthcare provider
- [ ] Increased mental well-being
- [ ] Increased social well-being
- [ ] Reduced stigma
- [ ] More freedom to live my life
- [ ] Other, please specify: ____
- [ ] ⊗ None of the above

For you, what would be the drawbacks of an injectable long-acting HIV PrEP (taken every 2 months)? Select all that apply.

- ☐ Pain from injection
- ☐ Scared of needles
- ☐ More frequent visits to my healthcare provider
- ☐ Concern about cosmetic side effect from receiving an injection
- ☐ Concern about other side effects
- ☐ Concern it might not work as well as pills
- ☐ I am worried about the cost
- ☐ Concerned whether injections will be effective for the full two months
- ☐ Decreased mental well-being
- ☐ Decreased social well-being
- ☐ Concern about missing two-monthly injections due to scheduling conflicts (e.g. travel)
- ☐ Increased stigma
- ☐ Other, please specify: ______________________________
- ☐ ⊗ None of the above

If both were available, what form of HIV PrEP would you prefer?

- ◯ I would prefer daily oral pills
- ◯ I would prefer an injection of long-acting medication every two months
- ◯ I don’t have a preference
- ◯ I am not interested in taking HIV PrEP regardless of the form
- ◯ Unsure

End of Block: HIV PrEP

Start of Block: HIV PrEP-HIRI

The following questions are from the "HIV Incidence Risk Index for Men Who Have Sex With Men” (HIRI-MSM)" which is used by clinicians to assess HIV risk. Canadian guidelines recommend anyone with a total score of greater than 10 to consider taking HIV PrEP.   The questions were primarily designed to be answered by cis men who have sex with cis men. We acknowledge this ignores the bodies and experiences of trans people and may not reflect the experiences of all 2S/LGBTQIA+ people.   **Do you want to answer these questions?**

- ◯ Yes
- ◯ No, please skip these questions

How old are you?

- ◯ < 18 years old (score of 0)
- ◯ 18-28 years (score of 8)
- ◯ 29-40 years (score of 5)
- ◯ 41-48 years (score of 2)
- ◯ 49 years or older (score of 0)

How many men have you had sex with in the last 6 months?

- ◯ 0-5 (score of 0)
- ◯ 6-10 (score of 4)
- ◯ More than 10 (score of 7)

In the last 6 months, did you have receptive anal sex with a man (you were the bottom) without a condom?

- ◯ Yes (score of 10)
- ◯ No (score of 0)

In the last 6 months, how many of your sex partners were men who were HIV-positive?

- ◯ 0 (score of 0)
- ◯ 1 (score of 4)
- ◯ More than 1 (score of 8)

In the last 6 months, how many times did you have insertive anal sex (you were the top) without a condom with a man who was HIV positive?

- ◯ 0-4 times (score of 0)
- ◯ 5+ times (score of 6)

In the last 6 months, have you used methamphetamines (crystal or speed)?

- ◯ Yes (score of 6)
- ◯ No (score of 0)

In the last 6 months, have you used poppers (amyl nitrate)?

- ○ Yes (score of 3)
- ○ No (score of 0)

Based on your responses, your HIRI score is ___. Canadian guidelines recommend anyone with a total score of greater than 10 to consider taking HIV PrEP. Talk to a healthcare provider if you are interested in HIV PrEP.

Have you had chlamydia, LGV, or gonorrhea in your bum in the PAST 12 MONTHS?
Canadian guidelines recommend that anyone diagnosed with one of these STIs in their bum in the past 12 months consider taking HIV PrEP.

- ○ Yes
- ○ No
- ○ I don't know

End of Block: HIV PrEP-HIRI

Start of Block: Healthcare

The questions in this section are about your healthcare.

Do you have a regular family doctor or nurse practitioner?

- ○ Yes
- ○ No

Do you have access to non-insured health benefits provided by the Federal government?
This may be accessed by having "status" (Registered/Treaty), sometimes referred to as having a status card or having treaty. We recognize that people may not be registered with a treaty for many reasons, even though they may be eligible.

- ◯ Yes
- ◯ No
- ◯ I don't know

Does your regular family doctor or nurse practitioner know about your sexual orientation?

- ◯ Yes
- ◯ No
- ◯ I don't know

Does your regular family doctor or nurse practitioner know about your gender identity/expression?

- ◯ Yes
- ◯ No
- ◯ I don't know

Have you EVER been vaccinated against the following?

	Yes	No	Unsure
COVID-19	☐	☐	☐
Hepatitis A Virus	☐	☐	☐
Hepatitis B Virus	☐	☐	☐
Human Papilloma Virus (HPV)	☐	☐	☐

SINCE MAY 2022, how many doses of mpox vaccine have you received?

- ○ 0
- ○ 1
- ○ 2
- ○ 3 or more
- ○ I don’t know

Have you EVER wanted or needed gender-affirming care (e.g., puberty blockers, hormones, or gender-affirming surgeries)?

- ○ Yes
- ○ No
- ○ I don’t know

Have you EVER tried to access gender-affirming care (e.g., puberty blockers, hormones, or gender-affirming surgeries)?

- ○ Yes
- ○ No
- ○ I don’t know

Were you able to access the gender affirming care (e.g., puberty blockers, hormones, or gender-affirming surgeries) you tried to access?

- ○ Yes, I was able to access all the gender affirming care I tried to access
- ○ Yes, I was able to access some but not all of the gender affirming care I tried to access
- ○ No
- ○ I don't know

Have you experienced any of the following barriers to your gender-affirming care? Select all that apply.

- ☐ Can't afford treatment (e.g., no health insurance, treatment not covered by insurance)
- ☐ Can't afford travel to treatment
- ☐ Feel unsafe to seek gender-affirming medical care due to transphobia in healthcare
- ☐ Feel unsafe to seek gender-affirming medical care due to racism/xenophobia in healthcare
- ☐ Feel unsafe to seek gender-affirming medical care due to transphobia or judgement from friends or family
- ☐ Don't have a family doctor or nurse practitioner with knowledge on gender-affirming care
- ☐ Don't have enough information about how to access gender-affirming care
- ☐ Denied because of my gender identity or expression
- ☐ Denied because of my weight
- ☐ Denied because of my mental health
- ☐ Denied because of my autism
- ☐ Denied because of my disability
- ☐ On a waitlist or long wait times
- ☐ Other, please specify: ______________________________
- ☐ ⊗ None of the above

We define chronic health conditions as those that meet all four of the following criteria:

1. Are physical, mental, cognitive, sensory, or psychological
2. Have lasted at least 1 year (or are expected to last at least 1 year)
3. Have an impact on your daily activities or "function", whether daily or episodic
4. Require some kind of supports such as medical care, assistive devices, or help from someone else.

We are using an expansive understanding of chronic health conditions to include conditions that are often under-represented, such as chronic pain, mental health conditions, HIV, being a cancer survivor, etc. Some people may identify as having a disability in addition to, or instead of, identifying as having a chronic health condition.

Based on the definition above, do you have a chronic health condition?

○ Yes

○ No

○ Unsure

End of Block: Healthcare

Start of Block: Mental Health

The questions in this section are about your mental health.

In general, how would you say your mental health is?

○ Excellent

○ Very good

○ Good

○ Fair

○ Poor

Over the last TWO WEEKS, how often have you been bothered by each of the following four problems?

	Not at all	Several days	More than half the days	Nearly every day
Little interest or pleasure in doing things	☐	☐	☐	☐
Feeling down, depressed, or hopeless	☐	☐	☐	☐
Feeling nervous, anxious, or on the edge	☐	☐	☐	☐
Not being able to stop or control worrying	☐	☐	☐	☐

How often do you feel…

	Hardly ever	Some of the time	Often
... that you lack companionship.	☐	☐	☐
...left out?	☐	☐	☐
...isolated from others?	☐	☐	☐

In the PAST 12 MONTHS, did you want help for any of the following? Select all that apply.

- [ ] Anxiety
- [ ] Body image
- [ ] Burnout
- [ ] Depression
- [ ] Eating disorder(s)
- [ ] Gender dysphoria
- [ ] Grief/loss
- [ ] Loneliness
- [ ] Navigating sexual orientation and identity
- [ ] Psychosis, hallucinations, delusions, or paranoia
- [ ] Relationship problems
- [ ] Self-harm
- [ ] Substance use
- [ ] Suicidal thoughts
- [ ] Trauma
- [ ] ⊗ None of the above

In the PAST 12 MONTHS, have you tried to use mental health counseling services?

- ○ Yes, and was able to access
- ○ Yes, but was not able to access
- ○ No, I did not try to access

In the PAST 12 MONTHS, which of the following resources have you gone to? Select all that apply.

- ☐ Elder
- ☐ Indigenous Knowledge keeper
- ☐ Psychiatrist
- ☐ Clinical psychologist
- ☐ Registered counsellor
- ☐ Peer counsellor/navigator
- ☐ Social worker
- ☐ Sex therapist / sexologist
- ☐ Regular family doctor or nurse practitioner (for mental health support)
- ☐ ⊗None of the above

End of Block: Mental Health

Start of Block: Discrimination and abuse

These questions are about potentially traumatic experiences and may be uncomfortable or triggering. They will ask you about discrimination and violence, including sexual abuse. You can choose to skip any question. **Would you prefer to skip this section of questions?**

- ◯ Yes
- ◯ No

In the PAST 12 MONTHS, have you experienced discrimination based on any of the following? Select all that apply.

- ☐ Age
- ☐ Body type/size
- ☐ Class or wealth
- ☐ Disabilities
- ☐ Gender
- ☐ Gender expression
- ☐ History of incarceration
- ☐ HIV PrEP status
- ☐ HIV status
- ☐ Housing status
- ☐ Immigration status
- ☐ Indigenous Identity
- ☐ Language and/or accent
- ☐ Race/ethnicity

- [ ] Sex work
- [ ] Sexual orientation
- [ ] Substance use
- [ ] Trans identity
- [ ] ⊗ None of the above

Have you EVER attended so-called conversion 'therapy' practices, by which we mean organized attempts (such as through counseling, psychological, behavioural, or faith-based rituals or practices) to change your sexual orientation or gender identity, or to deny or suppress 2S/LGBTQIA+ identities or non-normative gender expressions?

○ Yes

○ No

○ I'm not sure

Have you encountered an advertisement for conversion 'therapy' practices in Canada in the PAST 12 MONTHS?

○ Yes; please describe: ____

○ No

How would you describe your experience with conversion 'therapy' practices in the PAST 12 MONTHS? Select all that apply.

- ☐ I experienced 'conversion therapy'
- ☐ I think that I experienced 'conversion therapy', but I'm not sure
- ☐ I nearly experienced 'conversion therapy;' I considered going
- ☐ I nearly experienced 'conversion therapy', someone suggested that I go (e.g., a parent/guardian, religious leader, teacher, counselor, healthcare provider)
- ☐ I did not know 'conversion therapy' is still happening in Canada
- ☐ ⊗None of the above

Did you or any of your family members EVER attend a "residential school"? Select all that apply.

- ☐ Yes, I did
- ☐ Yes, at least one of my family members did
- ☐ ⊗No, neither myself nor any family member attended residential schools
- ☐ I don't know

Were you or any of your family members part of the Sixties Scoop? Select all that apply.

- ☐ Yes, I was part of the Sixties Scoop
- ☐ Yes, at least one of my family members was a part of the Sixties Scoop
- ☐ No, neither any family member nor myself were a part of the Sixties Scoop
- ☐ ⊗I don't know

Has a partner EVER done the following to you? If so, when was the last time?

	Yes, in the past 12 months	Yes, more than 12 months ago	No, Never
Insulted or verbally abused you	▢	▢	▢
Hit, kicked, slapped, or physically abused you	▢	▢	▢
Forced unwanted sex, raped, or sexually abused you	▢	▢	▢
Controlled or restricted your access to money	▢	▢	▢
Controlled or restricted your movements outside of the house	▢	▢	▢
Controlled or restricted your contact with your friends and family	▢	▢	▢

Has anyone EVER forced sex on you?

- ○ Yes, when I was younger than 18
- ○ Yes, when I was 18 or older
- ○ Yes, when I was both younger and older than 18
- ○ No, never

End of Block: Discrimination and abuse

Start of Block: Substance Use

The questions in this section are about your recent substance use.

Have you used any substances (including cigarettes, alcohol and cannabis) in the PAST 6 MONTHS? This does NOT include prescription medications.

- ○ Yes
- ○ No
- ○ Prefer not to answer

Why do you use substances? Select all that apply.

- [ ] To feel good/have a good time
- [ ] Because I like how they make me feel
- [ ] To feel better about bad things that happen to me
- [ ] To forget about bad things that happen to me
- [ ] To connect with others
- [ ] To improve my sex life
- [ ] To help with stress or anxiety
- [ ] To help with sadness or depression
- [ ] To help with pain
- [ ] To help me sleep
- [ ] Because my friends use them
- [ ] Because I am dependent (for example, to avoid withdrawal)
- [ ] Another reason not described above
- [ ] ⊗ None of the above

How often have you used the following substances in the PAST 6 MONTHS? Do not include substances or medications prescribed to you and used as prescribed.

	Never	Once in a while, not every week	Regularly, at least once a week	Daily
Alcohol (less than 5 drinks in one occasion)	☐	☐	☐	☐
Alcohol (5+ drinks in one occasion)	☐	☐	☐	☐
Tobacco (e.g., cigarettes or cigars)	☐	☐	☐	☐
Nicotine (e.g., vaping or e-cigarettes)	☐	☐	☐	☐
Cannabis, marijuana	☐	☐	☐	☐
Poppers/amyl nitrite	☐	☐	☐	☐
Cocaine	☐	☐	☐	☐
Ecstasy/MDMA	☐	☐	☐	☐
Erection drugs (e.g., Viagra, Cialis, TriMix)	☐	☐	☐	☐
GHB or GBL ("Gina", "G", "Water")	☐	☐	☐	☐
Crystal meth/Tina	☐	☐	☐	☐
Ketamine/Special K	☐	☐	☐	☐
Crack, Freebase	☐	☐	☐	☐
Tranquilizers or benzos (e.g., Valium, Xanax)	☐	☐	☐	☐
Amphetamine/Speed	☐	☐	☐	☐
Psychedelics (e.g., LSD, mescaline, acid, mushrooms)	☐	☐	☐	☐
Opioids (e.g., codeine, heroin, fentanyl, oxycocet/ “percs”, hydromorphone/ “dillies”)	☐	☐	☐	☐
Non-medical steroids	☐	☐	☐	☐
Other, please specify:	☐	☐	☐	☐

Have you EVER injected any substance or had someone else inject into you to get high? This does not include anabolic steroids, gender-affirming hormones or medicines used as prescribed for you. Select all that apply.

- ☐ Yes, in the past 6 months
- ☐ Yes, between 6 months and 5 years ago
- ☐ Yes, more than 5 years ago
- ☐ ⊗ No, never

Which substances have you injected in the PAST 6 MONTHS? Select all that apply.

- ☐ Crystal meth/Tina
- ☐ Cocaine
- ☐ Opioids (e.g., heroin/"smack",fentanyl)
- ☐ Ketamine/Special K
- ☐ Amphetamine/Speed
- ☐ Ecstasy/"E"/MDMA
- ☐ Crack
- ☐ Other, please specify below:
  ____
- ☐ I've injected drugs, but I don't know which drugs

In the PAST 6 MONTHS, when you injected drugs, did you use needles and/or syringes that had already been used by someone else?

- ○ Yes
- ○ No
- ○ Don’t know
- ○ Prefer not to answer

Have you EVER used substances to make sex more intense, less inhibited, or last longer? Select all that apply.

- ☐ Yes, in the past 6 months
- ☐ Yes, more than 6 months ago
- ☐ ⊗ No, never

What substances did you use to make sex more intense, less inhibited, or last longer in the PAST 6 MONTHS? Select all that apply.

- ☐ Alcohol (5+ drinks in one occasion)
- ☐ Alcohol (less than 5 drinks in one occasion)
- ☐ Tobacco (e.g., cigarettes or cigars)
- ☐ Nicotine (e.g., vaping or e-cigarettes)
- ☐ Cannabis, marijuana
- ☐ Poppers/amyl nitrite
- ☐ Cocaine
- ☐ Ecstasy/E/MDMA

- ☐ Erection drugs (e.g., Viagra, Cialis)
- ☐ GHB or GBL ("Gina", "G", "Water")
- ☐ Crystal meth/Tina/T
- ☐ Ketamine/Special K
- ☐ Crack, Freebase
- ☐ Tranquilizers or benzos (e.g., Valium, Xanax)
- ☐ Amphetamines (e.g., speed, mephedrone)
- ☐ Psychedelics (e.g., LSD, mescaline, acid, mushrooms)
- ☐ Opioids (e.g., codeine, heroin, fentanyl, oxycocet, hydromorphone/"dillies")
- ☐ Non-medical steroids (e.g., anabolic steroids)
- ☐ Other, please specify ______________________________
- ☐ ⊗ None of the above

In the PAST 6 MONTHS, have you used any of the following? Select all that apply.

- ☐ Needle exchange
- ☐ Harm reduction supplies (e.g., free pipes, straws)
- ☐ Supervised injection, inhalation or consumption site
- ☐ Naloxone/NARCAN was used on me
- ☐ I used Naloxone/NARCAN on someone else

☐ Detox or drug treatment facility

☐ Sweat Lodge or other cultural traditions

☐ Peer support services

☐ Other, please specify:
____

☐ ⊗None of the above

End of Block: Substance Use

Start of Block: Gambling

The next questions are about gambling practices, including in-person gambling (e.g., casinos) and online gambling (websites or apps on a computer, smartphone, or tablet). These questions specifically refer to gambling with real money, where you can win or lose actual cash.   We ask these questions without any judgment, aiming to better understand the diverse gambling practices within our communities. Your honest responses will help us identify how we can best support everyone's needs.

Which of the following online gambling activities have you participated in over the PAST 12 MONTHS? Select all that apply.

- ☐ Purchased lottery tickets online such as 6/49 and Lotto Max
- ☐ Purchased or played online instant lottery tickets, such as scratch, break-open, or pull-tabs
- ☐ Played online traditional bingo
- ☐ Played online slots machine
- ☐ Played, bet or spent money on online casino table games (like poker, blackjack, Baccarat or roulette)
- ☐ Placed online bets on horse races
- ☐ Placed online bets on sports
- ☐ Placed online bets on E-Sports (video game competitions)
- ☐ Engaged in online betting on financial markets (FOREX, binary options)
- ☐ Other online gambling activity, please specify: ____________________
- ☐ ⊗ I have not participated in any gambling activities

In the PAST 12 MONTHS, how often have you participated in online gambling activities?

- ○ Less than once a month
- ○ Once a month
- ○ Two or three times a month
- ○ Once a week
- ○ Several times a week

Which of the following in-person gambling activities have you participated in over the PAST 12 MONTHS? Select all that apply.

- ☐ Purchased in-person/physical lottery tickets such as 6/49 and Lotto Max
- ☐ Purchased or played in-person/physical instant lottery tickets, such as scratch, break-open or pull-tabs
- ☐ Played Drag Bingo (Bingo hosted by Drag queens, for charity or not)
- ☐ Played Traditional Bingo
- ☐ Played slots machine (found in Casinos)
- ☐ Played video lottery in gay or queer venues (bars and restaurants)
- ☐ Played video lottery in regular restaurants and bars
- ☐ Played, bet or spent money on casino table games (like poker, blackjack, Baccarat or roulette)
- ☐ Placed bets in-person on horse races
- ☐ Placed bets in-person on sports
- ☐ Placed bets in-person on E-Sports in-person (video game competitions)
- ☐ Engaged in betting (in-person) on financial markets (FOREX, binary options)
- ☐ Other in-person gambling activity, please specify: ____________________
- ☐ ⊗ I have not participated in any gambling activities

In the PAST 12 MONTHS, how often have you participated in in-person gambling activities?

- ◯ Less than once a month
- ◯ Once a month
- ◯ Two or three times a month
- ◯ Once a week
- ◯ Several times a week

In the PAST 12 MONTHS, approximately how much money have you spent on gambling activities (both online and in-person) a month?

- ◯ $0 per month
- ◯ $1 to $49 per month
- ◯ $50 to $100 per month
- ◯ $101 to $500 per month
- ◯ $501 to $1000 per month
- ◯ More than $1000

In the PAST 12 MONTHS, approximately how much time have you usually spent on gambling activities (both online and in-person) in a month?

- ◯ Less than 1 hour per month
- ◯ 1 to 5 hours per month
- ◯ 6 to 10 hours per month
- ◯ 11 to 15 hours per month
- ◯ More than 15 hours per month

Some of the following questions may not seem relevant to you, but they are important for understanding different experiences and needs. Your responses help provide a complete picture of our communities. Thinking of the last 12 months...

	Never	Sometimes	Most of the time	Almost always
How often have you bet more than you could really afford to lose?	☐	☐	☐	☐
How often have you needed to gamble with larger amounts of money to get the same feeling of excitement?	☐	☐	☐	☐
When you gambled, how often did you go back another day to try to win back the money you lost?	☐	☐	☐	☐
Have you borrowed money or sold anything to get money to gamble?	☐	☐	☐	☐
How often has your gambling caused any financial problems for you or your household?	☐	☐	☐	☐
How often have you felt that you might have a problem with gambling?	☐	☐	☐	☐
How often has your gambling caused you any health problems, including stress or anxiety?	☐	☐	☐	☐
How often have people criticized your betting or told you that you had a gambling problem, regardless of whether or not you thought it was true?	☐	☐	☐	☐
How often have you felt guilty about the way you gamble or what happens when you gamble?	☐	☐	☐	☐

How important do you think it is to have gambling support services specifically tailored for the 2S/LGBTQIA+ community?

- ◯ Very important
- ◯ Important
- ◯ Not important
- ◯ Not important at all

If 2S/LGBTQIA+ specific gambling support services were available, how likely would you be to use them?

- ◯ Very likely
- ◯ Somewhat likely
- ◯ Somewhat unlikely
- ◯ Very unlikely

End of Block: Gambling

Start of Block: Community Connection

The questions in this section are about community and your social connections.

How many people can you count on for support if you need help or if something goes wrong?

- ◯ 1 person
- ◯ 2-3 people
- ◯ 4-6 people
- ◯ 7-9 people
- ◯ 10+ people
- ◯ No one

How connected do you feel with other people in 2S/LGBTQIA+ communities?

- ◯ Not at all connected
- ◯ A little connected
- ◯ Moderately connected
- ◯ Very connected
- ◯ Extremely connected

How included do you feel in 2S/LGBTQIA+ communities?

- ◯ Not at all included
- ◯ A little included
- ◯ Moderately included
- ◯ Very included
- ◯ Extremely included

How important is it to you to be involved in 2S/LGBTQIA+ communities?

- ◯ Not at all important to me
- ◯ A little important
- ◯ Moderately important
- ◯ Very important
- ◯ Extremely important to me

Thank you for completing our survey!

Your feedback is important in shaping the questions we will ask in future Sex Now surveys! This was the first Sex Now that was open to all 2S/LGBTQIA+ people. We want to learn how we can enhance your experience with our survey in the future. **Is there anything you would suggest adding or changing in the next Sex Now survey?**

____

____

____

____

____

End of Block: Community Connection
